# Molecular and genetic characterization of partial masculinization in embryonic ovaries grafted into male nude mice

**DOI:** 10.1371/journal.pone.0212367

**Published:** 2019-03-06

**Authors:** Kento Miura, Kyoko Harikae, Mayu Nakaguchi, Kenya Imaimatsu, Ryuji Hiramatsu, Ayako Tomita, Yoshikazu Hirate, Masami Kanai-Azuma, Masamichi Kurohmaru, Atsuo Ogura, Yoshiakira Kanai

**Affiliations:** 1 Department of Veterinary Anatomy, The University of Tokyo, Bunkyo-ku, Tokyo, Japan; 2 RIKEN BioResovurce Research Center, Tsukuba, Ibaraki, Japan; 3 Center for Experimental Animals, Tokyo Medical and Dental University, Bunkyo-ku, Tokyo, Japan; 4 RIKEN Cluster for Pioneering Research, Wako, Saitama, Japan; 5 Graduate School of Life and Environmental Sciences, University of Tsukuba, Tsukuba, Ibaraki, Japan; University of Hyderabad, INDIA

## Abstract

In most of mammalian embryos, gonadal sex differentiation occurs inside the maternal uterus before birth. In several fetal ovarian grafting experiments using male host mice, an experimental switch from the maternal intrauterine to male-host environment gradually induces partial masculinization of the grafted ovaries even under the wild-type genotype. However, either host-derived factors causing or molecular basis underlying this masculinization of the fetal ovaries are not clear. Here, we demonstrate that ectopic appearance of SOX9-positive Sertoli cell-like cells in grafted ovaries was mediated by the testosterone derived from the male host. Neither *Sox8* nor *Amh* activity in the ovarian tissues is essential for such ectopic appearance of SOX9-positive cells. The transcriptome analyses of the grafted ovaries during this masculinization process showed early downregulation of pro-ovarian genes such as *Irx3*, *Nr0b1*/*Dax1*, *Emx2*, and *Fez1*/*Lzts1* by days 7–10 post-transplantation, and subsequent upregulation of several pro-testis genes, such as *Bhlhe40*, *Egr1/2*, *Nr4a2*, and *Zc3h12c* by day 20, leading to a partial sex reversal with altered expression profiles in one-third of the total numbers of the sex-dimorphic pre-granulosa and Sertoli cell-specific genes at 12.5 dpc. Our data imply that the paternal testosterone exposure is partially responsible for the sex-reversal expression profiles of certain pro-ovarian and pro-testis genes in the fetal ovaries in a temporally dependent manner.

## Introduction

In mouse sex differentiation, both testicular Sertoli cells and ovarian granulosa cells develop from common supporting cell precursors in the genital ridges [1,2]. In XY male mice, SRY, sex-determining region on Y chromosome, directly upregulates an autosomal SRY-related HMG box (*Sox*) gene, *Sox9*, in bipotential supporting cells from 11.0 days post-coitum (dpc) [3–7]. SOX9 subsequently induces the *Sox8* expression [8,9], in addition to activating several male-specific signaling factors, including FGF9 [10–12]. After the cessation of transient SRY expression, *Sox9* and *Sox8* cooperatively maintain the function of Sertoli cells during the later stages [13–16]. In the absence of *Sry*, bipotential supporting cells differentiate into pre-granulosa cells from approximately 11.5 dpc in mouse XX embryos [17]. After 12.0 dpc, pre-granulosa cells start to express FOXL2 (forkhead box L2; an ovarian-specific transcriptional factor [18]), which is involved in ovarian development and maintenance, along with high levels of WNT4/RSPO1/β-catenin signaling [15,19–22]. In addition to such female pathways of pro-ovarian genes, estrogen also plays crucial roles in the maintenance of granulosa cells after birth. For example, *Esr1/2* (*estrogen receptor-1/-2*) double-null females develop ectopic SOX9-positive Sertoli cell-like (Sertoli-like) cells during the initial round of folliculogenesis, leading to ovotestis formation upon reaching the adult stages [23,24]. In most cases of the female-to-male sex reversal by a loss-of-function mutation of such pro-ovarian genes, masculinized phenotypes become evident only after birth [25]. This suggests possible roles of the intrauterine maternal environment in the maintenance of fetal ovarian differentiation and development. However, the protective role of intrauterine maternal environment in ovarian differentiation and sex reversal remains unclear at present.

Freemartin syndrome causes infertility in a female cattle twin born with a male twin [26,27]. Since the female bovine fetus shares a blood supply with the male fetus, some circulating factors derived from the male twin (e.g., testosterone and AMH [anti-Müllerian hormone, a TGF-beta superfamily member]) cause masculinization of the genital organs of the female twin, including testis-like structures with SOX9-positive Sertoli-like cells in some severe cases [28]. Such masculinization is commonly seen in other vertebrates including fish, frog and bird, in which sex hormone treatment could induce a complete sex reversal in their gonads [29–32].

Similar to freemartin ovaries, fetal ovarian grafts under the kidney capsules of adult male mice undergo a partial masculinization [33,34], together with ectopic appearance of SOX9-positive Sertoli-like cells approximately 15–20 days post-transplantation [17,35]. Interestingly, our previous study using the fetal ovaries carrying an inducible SRY transgene demonstrated that most of differentiated granulosa cells in these ovarian grafts re-acquire the SDSI (SRY-dependent SOX9 inducibility)-positive state (i.e., a “ready-to-go” state of *Sox9* transcription) during 7–10 days post-transplantation [4,17], showing a similar bipotential state of the pre-granulosa cells at 11.0–11.5 dpc. Moreover, such ovarian grafts develop ectopic formation of testis cord-like structures and subsequent appearance of SOX9-positive Sertoli-like cells on the mesonephric side by day 20 post-transplantation. These findings suggest that a switch from the maternal to male-host environment gradually induces partial masculinization of fetal ovaries even under the wild-type genotype. However, either host-derived factors causing or the molecular basis underlying the masculinization of fetal ovarian grafts in the male-host environment is not clear at present.

In the present study, we examined the roles of host-derived testosterone and donor-derived *Sox8* and *Amh* activity in the partial masculinization of fetal ovaries in the male-host environment. We also examined temporal changes in the gene expression profiles of grafted fetal ovaries during the masculinization process in male nude mice and compared these expression profiles with those from XY/XX embryos during the normal testicular/ovarian differentiation process.

## Results

### Partial masculinization of fetal ovarian grafts mediated partly by the testosterone derived from male hosts

In fetal ovaries grafted under the kidney capsules of adult male mice (XY-host), the ovarian transplants undergo follicular degeneration by day 10 post-transplantation in which cord-like structures with SOX9-positive Sertoli-like cells appear in the gonadal parenchyma on day15–20 post-transplantation [17,35].

**Fig 1:**
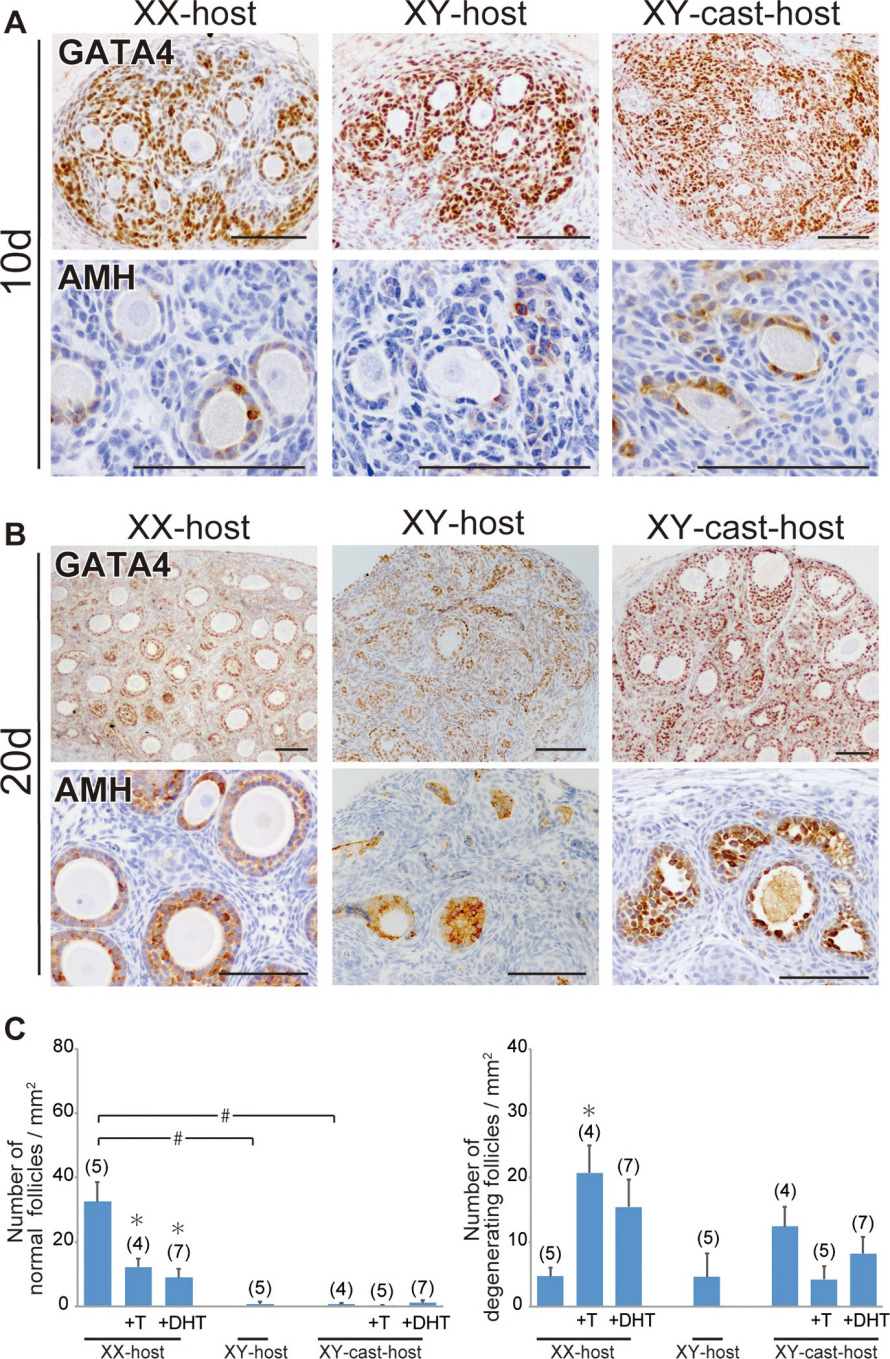
Repression of initial follicular growth in ovarian grafts under androgen-excess host conditions. **(A, B)** Anti-GATA4 and AMH immunostaining of wild-type ovarian tissues grafted into intact male (XY-host), intact female (XX-host) and castrated male (XY-cast-host) hosts treated with or without testosterone (T), or dihydrotestosterone (DHT). The lower magnified images of GATA4-positive gonadal areas are shown in upper plates in A and B. AMH-positive healthy primary, secondary, and antral follicles were detected in ovaries grafted into female hosts (lower plate in B). **(C)** Bar graphs indicate the relative numbers of normal healthy follicles (left) and degenerating follicles (right). The data are expressed as means ± SEM (*p<0.05 as compared with non-treated host value in each host group; #p<0.05 as compared between two groups, Steel's test). Each number in parentheses indicates the number of explants used in each host. Scale bars, 100 μm for (A, B).

First, to examine the contribution of the male-host environment to the follicular degeneration, we transplanted fetal ovaries (wild-type, 13.0 dpc) under the kidney capsules of intact female (XX) or male (XY) nude mice, and then conducted immunohistochemical staining with anti-AMH (a marker for both Sertoli cells and mature granulosa cells of growing follicles) and anti-GATA4 (GATA binding protein 4; a marker for all gonadal somatic cell types) antibodies (Fig 1). In ovarian transplants grafted into XX-host, the primary and secondary AMH-positive follicles were well-developed by day 10 post-transplantation, although some degenerating follicles with either atretic oocytes or disorganized granulosa cells were present (Fig 1A). On day 20, AMH-positive secondary and antral follicles were found in the GATA4-positive gonadal parenchyma, indicative of the proper initial round of follicular growth in ovarian explants grafted into intact female nude mice (“XX-host” in Fig 1B). In the ovarian transplants grafted into XY-host, degenerating follicles were evident by day 10 post-transplantation (Fig 1A), leading to the subsequent loss of the ovarian follicles by day 15–20 post-transplantation (Fig 1B). Next, we examined the contribution of the testis-derived hormones on follicular degeneration of the ovarian grafts in XY-host, we transplanted fetal ovaries into castrated male (XY-cast) nude mice. The defective follicular development in XY-host appears to be partially rescued by the castration treatment of host male mice, but no healthy follicles, albeit of the remaining large degenerative ones, was detected in ovarian explants grafted into XY-cast-host (“XY-cast-host” in Fig 1B). To examine the effect of testis-derived androgen on follicular development in the ovarian explants, we next transplanted fetal ovaries into XX- and XY-cast-hosts treated with or without testosterone (T) or dihydrotestosterone (DHT; non aromatizable androgen), and then examined morphometrically the appearance incidences of the healthy follicles or degenerative follicular structures (i.e., non-healthy follicles) in each host group (Fig 1C). In XX-host, T or DHT treatment decreased the number of healthy follicles and increased that of degenerative ones in their ovarian grafts (most left three bars in Fig 1C). In three XY-cast groups, as well as XY-host, few healthy follicles was detected in the ovarian grafts, possibly due to a lack of host ovaries as the primary source of estrogens. The relative number of degenerative follicles in the ovarian grafts also showed similar moderate levels among XY-host and XY-cast-host treated with or without T/DHT (most right three bars in Fig 1C). These results suggest the positive contribution of androgens to the degeneration of normal follicles in the ovarian grafts in the XX-host environment, but no significant influences of androgens on the development/degeneration of the ovarian follicles in the non-female (i.e., presumptive low-estrogen) host environment. In addition, in each male host group, the presumptive androgen levels (i.e., relative weight of seminal vesicles [36]) compared to control (intact) XY-host showed approximately 11% in XY-cast-host, 59% in XY-cast-host +T, or 54% in XY-cast-host +DHT, respectively (as set 100% in control XY-host; see S1 Fig).

**Fig 2:**
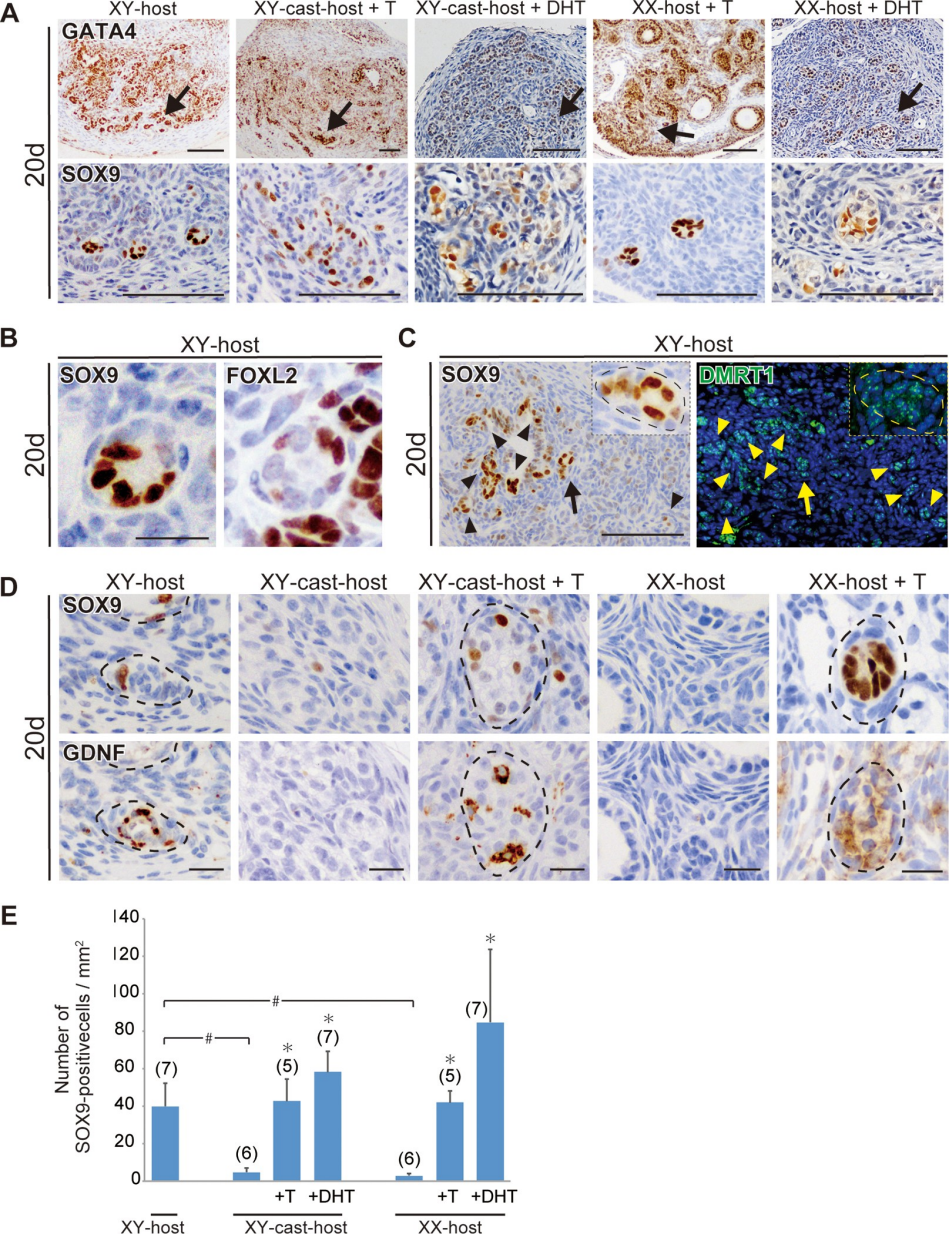
Ectopic appearance of SOX9-posititve Sertoli-like cells in ovarian grafts under androgen-excess host conditions. **(A–D)** Anti-GATA4, SOX9, FOXL2, DMRT1, or GDNF immunostaining of wild-type ovarian tissues grafted into XY-, XX-, and XY-cast-hosts treated with or without T or DHT on days 20 post-transplantation. The lower magnified images of GATA4-positive gonadal areas are shown in upper plates in a. Normal follicular structures were mostly absent, and tubular structures containing ectopic SOX9-positive cells were evident near the edge of ovaries grafted into XY-host and T/DHT-treated XX- and XY-cast-hosts on day 20 post-transplantation (arrows in A). The ectopic SOX9-positive cells are FOXL2-negative (B), and they are found in the DMRT1-positive/GDNF-positive tubular region (C, D). In c, the inset shows high-magnification images of tubular structures indicated by arrows. **(E)** Bar graphs indicate the SOX9-positive cells per gonadal area (mm^2^). The data are expressed as means ± SEM (*p<0.05 as compared with non-treated host value in each host group; #p<0.05 as compared between two groups, Steel's test). Each number in parentheses indicates the number of explants used in each host. Broken lines indicate the border of tubular structures in c and e. Scale bars, 100 μm for (A, C); 20 μm for (B, D).

Next, we examined the effect of XY-host-derived androgen on the ectopic appearance of SOX9-positive Sertoli-like cell in ovarian transplants. In the ovarian explants grafted into control XY-host, any SOX9-positive signals were not detectable throughout the ovarian parenchyma on day 10 post-transplantation, but ectopic SOX9-positive Sertoli-like cells were found to appear in the testis cord-like structure near the edge of the GATA4-positive gonadal parenchyma by day 20 (Fig 2A–2C; S2 Fig), which may correspond to the ovarian medullary region adjacent to the mesonephros (see Fig 6 in Harikae et al. [17]). The ectopic SOX9-positive cells in ovarian explants at day 20 are negative for anti-FOXL2 staining (a female-specific marker of both granulosa and theca cells) (Fig 2B), and frequently found in the DMRT1 (a male-specific maker of germ and supporting cells after 13.5 dpc [37,38])-positive and GDNF (a Sertoli-specific functional marker and a niche factor for spermatogonial stem cells [39,40])-positive tubular structures (Fig 2B–2D). In fetal ovaries transplanted into XY-cast-host, only a few SOX9-positive Sertoli-like cells were identified by serial sectioning analysis in XY-cast-host as well as those in XX-host with normal follicular growth (Fig 2D). In contrast, fetal ovaries transplanted into XY-cast-host +T/DHT clearly exhibited ectopic appearance of SOX9-positive cells in GDNF-positive tubular structures in the presumed medullary regions, similar to the masculinizing phenotype of ovarian transplants grafted into XY-host (Fig 2A and 2D). Moreover, ectopic appearance of SOX9-positive cells in tubular structures were also high frequently detectable even in XX-host treated with T/DHT (Fig 2A and 2D). Quantitative data also confirmed the significant increases of the relative SOX9-positive cell number by T/DHT treatment in either XY-cast- or XX-hosts (Fig 2E). These findings, therefore, suggest that testosterone actions may contribute to the appearance of SOX9-positive Sertoli-like cells in ovarian grafts in this partial masculinization model.

### Potential roles of *Sox8* and *Amh* in the follicular degeneration in ovarian grafts in the male-host environment

In the testis differentiation of *Rspo1*/*Sox9*-double null XX mice, *Sox8* appears to act as a pro-testis gene that induces male differentiation without any actions of SRY and SOX9 [19,41,42]. AMH also acts as a masculinizing factor in the female embryos of several mammals, including those with freemartin syndrome (Bogdanova et al. [43]; French et al. [44] and references therein). In previous *in vivo* and *in vitro* experiments, excessive AMH exposure was able to induce seminiferous cord-like structures in fetal rodent ovaries [45,46]. Based on these reports, we examined the potential contributions of these two pro-testis genes, *Sox8* and *Amh*, to partial masculinization of fetal ovaries in the present model.

**Fig 3:**
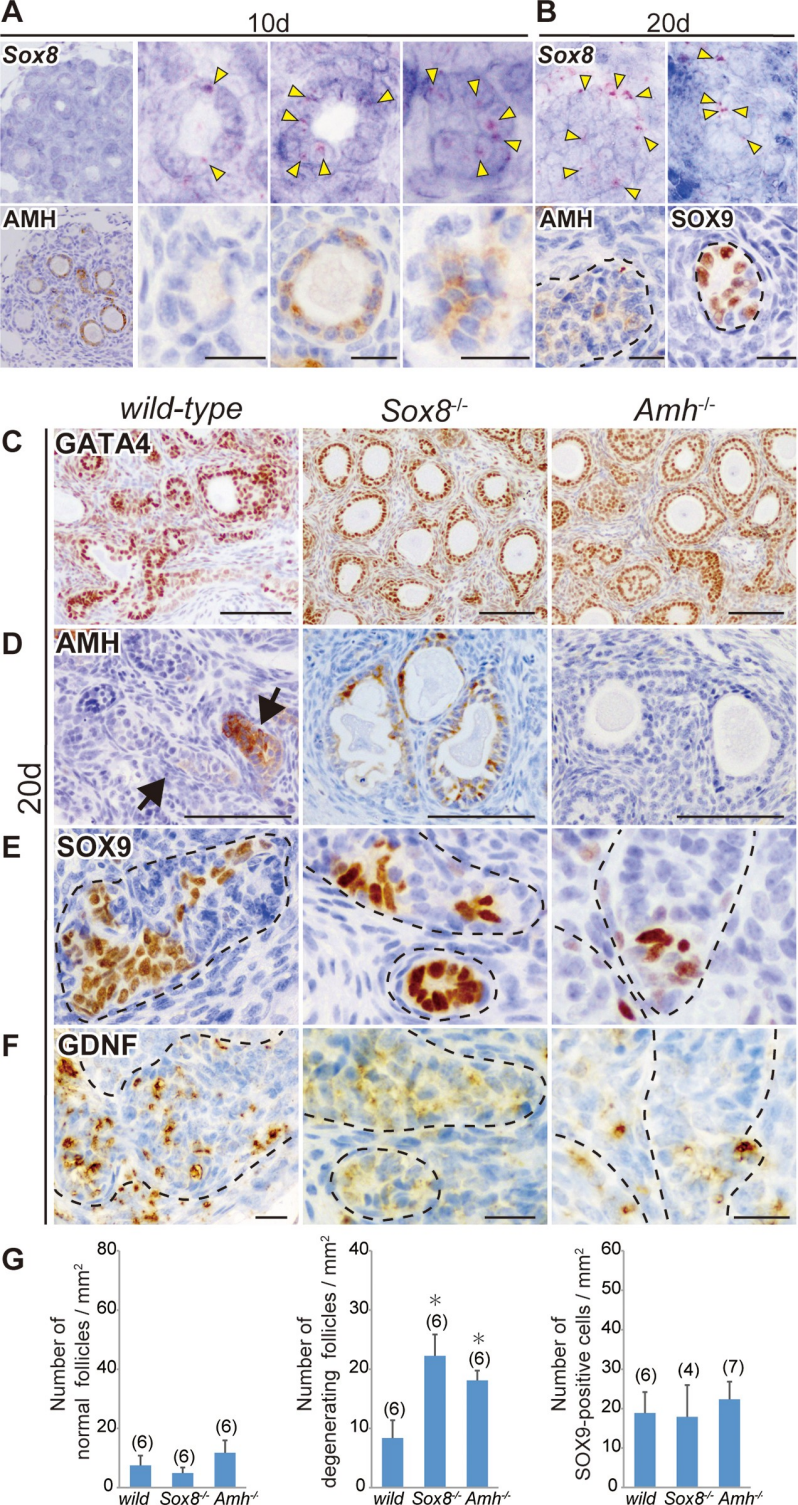
*Sox8* expression in AMH-positive and -negative degenerating follicles and masculinized phenotypes of the *Sox8*-null and *Amh*-null ovaries transplanted into male host mice. **(A, B)** *In situ* hybridization using a *Sox8* antisense probe and anti-AMH immunostaining of two serial ovarian tissue sections grafted into XY-host on days 10 (A; The most left panels are lower and the others are higher magnified images) and 20 (B) post-transplantation. *Sox8*-positive signals (yellow arrowheads) were detected in the granulosa cells of AMH-negative and -positive degenerating follicles on day 10 post-transplantation, as well as in tubular structures, including SOX9-positive Sertoli-like cells on day 20 post-transplantation. **(C–F)** Anti-GATA4, AMH, SOX9, or GDNF immunostaining of wild-type, *Sox8*-null (*Sox8*^-/-^), or *Amh*-null (*Amh*^-/-^) ovarian tissues grafted into XY-host on day 20 post-transplantation. The gonadal area is identified by GATA4 staining (C). Degenerating follicles were frequently seen in either *Sox8*-null or *Amh*-null grafted ovaries even at this stage (C, D). In contrast, the tubular structure formation (arrows or broken outlines) and ectopic appearance of SOX9-positive Sertoli-like cells in GDNF-positive tubular structures are found in all three types of grafted ovaries (E, F). (G) Bar graphs indicate the relative numbers of normal healthy follicles (left), degenerating follicles (center), and SOX9-positive cells (right) per gonadal area (mm^2^) in wild-type, *Sox8*-null, or *Amh*-null grafts in male host mice. The *Sox8*-null or *Amh*-null ovarian explants exhibited a large number of degenerating follicles even on day 20 post-transplantation, but no changes in the number of SOX9-positive cells were detected among the three genotypes. The data are expressed as means ± SEM (*p<0.05 as compared with wild-type host value, Dunnett's test). The numbers in parentheses indicate the number of explants used in each host. Broken lines indicate the border of tubular structures. Scale bars, 20 μm for (A, B, F); 100 μm for (C, D).

To examine the spatiotemporal patterns of *Sox8* expression in ovarian grafts, we used serial sections of ovarian grafts on days 10 and 20 post-transplantation for *in situ* hybridization using a *Sox8* antisense probe (Fig 3A and 3B). *Sox8* signals were detectable in the granulosa cells of both AMH-negative and -positive degenerating follicles on day 10 post-transplantation into XY-host (Fig 3A). On day 20 post-transplantation into XY-host, *Sox8*-positive signals were frequently observed in tubular structures, including SOX9-positive Sertoli-like cells near the presumptive medullary region (Fig 3B), suggesting the widely expressed *Sox8* profiles in some granulosa cells of the degenerating follicles and tubular structures in the ovarian grafts during the masculinization processes.

Next, to examine the roles of *Sox8* and *Amh* in the masculinization of ovarian grafts, we established *Sox8*-null (*Sox8*^-/-^) and *Amh*-null (*Amh*^-/-^) lines using the CRISPR/Cas9 system (S3 and S4 Figs). *Sox8*^-/-^ or *Amh*^-/-^ fetal ovaries were transplanted into XY-host, and their masculinized phenotypes were examined on day 20 post-transplantation. In both *Sox8*^-/-^ and *Amh*^-/-^ fetal ovarian grafts, degenerating follicles were detected along with typical testis cord-like structures on day 20 post-transplantation (Fig 3C–3F). There were no appreciable differences in the number of normal follicles among all three kinds of grafted ovaries (left graph in Fig 3G). The number of degenerating follicles was significantly higher in ovarian explants on day 20 in both *Sox8*^-/-^ and *Amh*^-/-^ ovarian explants compared with wild-type ovarian explants (Fig 3C and 3D, center graph in G), in which degenerating follicles with large oocytes were frequently seen in both two mutant explants (“*Sox8*^-/-^*”* and “*Amh*^-/-^*”* in Fig 3C and 3D). Since no appreciable alteration in normal follicular number was found in ovarian grafts of each mutant (left graph in Fig 3G), such increased atretic follicles on day 20 post-transplantation may reflect the gradual degeneration of ovarian follicles and/or the resistance of follicular degeneration into the tubular structure without any oocyte in each mutant. However, despite such protective follicular degeneration in each mutant, all three types of grafted ovaries exhibited ectopic SOX9-positive Sertoli-like cells in the GDNF-positive tubular structures, and there were no appreciable differences in the relative number of SOX9-positive Sertoli-like cells (Fig 3E and 3F, right graph in G). These data suggest that neither *Sox8* nor *Amh* activity in the ovarian tissues is essential for such ectopic appearance of SOX9-positive cells in ovarian grafts, suggesting a potential involvement of previously unknown factors/mechanisms in the present masculinization model.

### Global temporal changes in pre-granulosa or Sertoli cell-specific genes in ovarian grafts in the male-host environment

**Fig 4:**
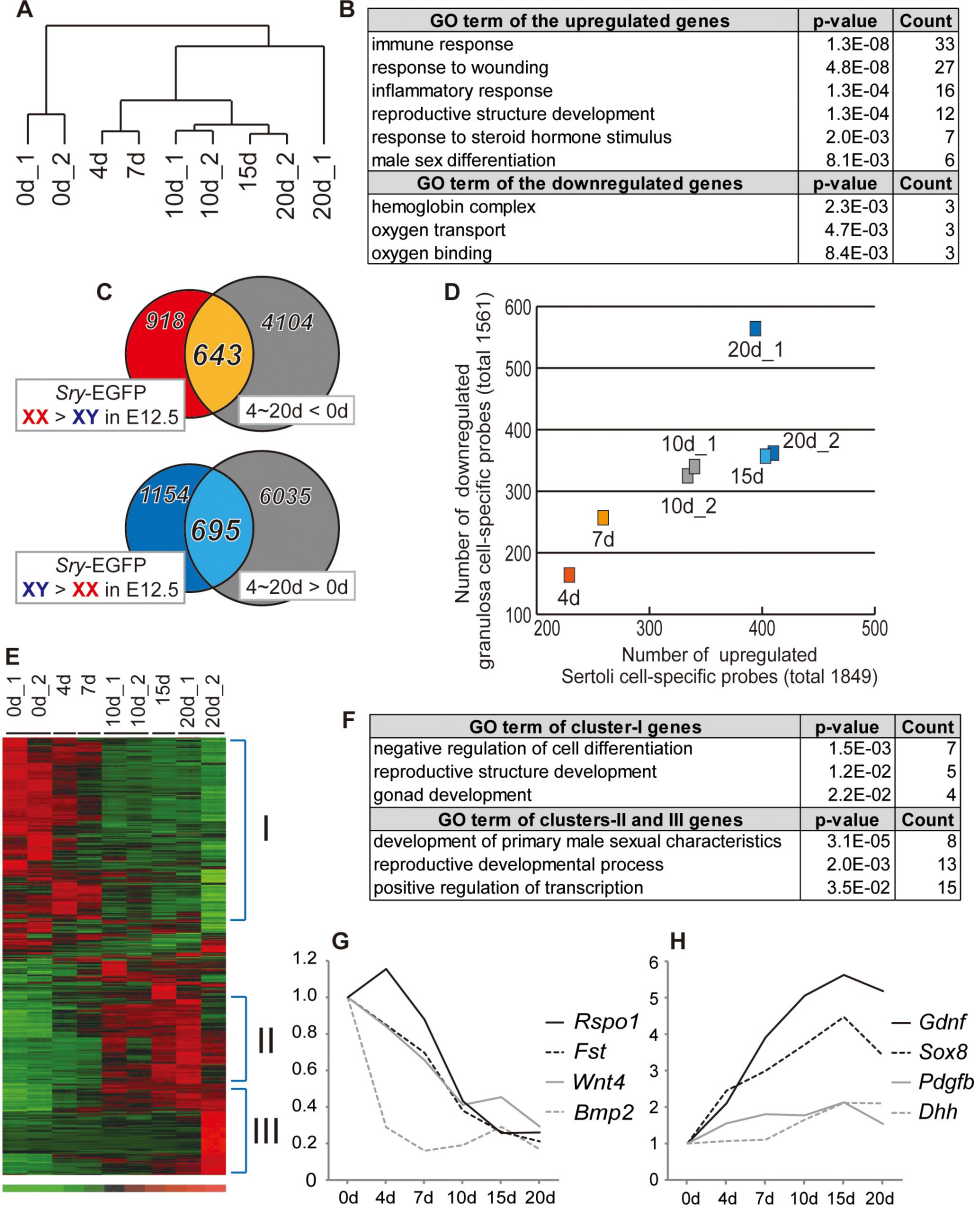
Temporal changes in the expression levels of pre-granulosa or Sertoli cell-specific genes in ovarian grafts in the male-host environment. **(A)** Hierarchical clustering of microarray data obtained from the grafted ovaries on days 0, 4, 7, 10, 15, and 20 post-transplantation, based on all 45,101 probes in the Mouse Genome 430 2.0 GeneChip array. Duplicate raw data were generated from transplants on days 0, 10, and 20 post-transplantation. **(B)** GO terms (p<0.01) for 241 down/576 upregulated genes (|fold| > 2, false discovery rate [q] < 0.3) in grafted ovaries on day 20 post-transplantation. **(C)** Venn diagrams showing 643 pre-granulosa (orange) and 695 Sertoli cell (blue)-specific gene probes selected from among the altered gene probes at each stage by comparing data from Sertoli cells versus pre-granulosa cells at 12.5 dpc using a published dataset [47] (right circles). **(D)** Scatterplot showing the number of down/upregulated pre-granulosa/Sertoli cell-specific genes in the grafted ovaries. Each point on the graph shows the grafted ovaries on each day post-transplantation. The vertical or horizontal lines show the total numbers of downregulated pre-granulosa or upregulated Sertoli cell-specific genes (|fold| > 2, p<0.05) at 12.5 dpc from the published dataset [42]. **(E)** Heatmap showing relative changes in expression levels of the 525 pre-granulosa (containing cluster I) and 402 Sertoli cell-specific genes (containing clusters II/III) in grafted ovaries (signal intensity: high [red], medium [black] to low [green]). **(F)** GO terms (p<0.05) for cluster I and clusters II/III genes in the ovarian grafts. **(G, H)** Line graphs show the fold expression changes for several key pre-granulosa (G) and Sertoli cell-specific genes (H) post-transplantation. The expression levels on day 0 post-transplantation were set as 1.0 on the y-axis.

To characterize global changes in gene expression associated with partial masculinization, we performed microarray analyses using germ cell-depleted grafted ovaries (i.e., ovarian tissues prepared from busulfan-pretreated embryos) before and after transplantation into XY-host and examined temporal changes in the expression profiles of ovarian somatic cells from 4 to 20 days post-transplantation. According to hierarchical clustering, the grafted ovaries exhibited gene expression profiles that differed from those of 13.0 dpc ovaries pre-transplantation (Fig 4A). GO analyses of the significantly upregulated genes on days 10 and 20 post-transplantation identified associations with functional categories including “reproductive structure development”, “response to steroid hormone stimulus”, and “male sex differentiation”, including *Ar* (*androgen receptor*), *Tgfbr*2 (*transforming growth factor*, *beta receptor II*), etc. (Fig 4B, S1 Table). This may be partly due to the exposure of ovarian transplants to follicle-derived AMH and host-derived testosterone during the masculinization process. Moreover, most of the other GO terms assigned to the significantly altered genes were associated with side effects from the transplantation experiment. For example, some terms were associated with inflammation (e.g., “immune response”, “response to wounding”, and “inflammatory response”) among upregulated genes or with blood-related changes from fetal females to adult males (e.g., “hemoglobin complex”, “oxygen binding”, and “oxygen transport”) among the downregulated genes (Fig 4B). These results suggest that a considerable number of the altered transcripts were associated with side effects from transplantation, such as injury, wound healing, and altered blood supply.

Next, to analyze the genes that were not associated with these side effects, we selected genes shared between the 1,561 pre-granulosa and 1,849 Sertoli cell-specific genes in fetal ovaries and testes at 12.5 dpc (Fig 4C, S2 Table). Among the 4,747 downregulated and 6,730 upregulated genes in ovarian transplants, 41.2% (643/1,561 gene probes) of the pre-granulosa cell-specific genes and 37.6% (695/1,849 gene probes) of the Sertoli cell-specific genes were down- or upregulated by day 20 post-transplantation (Fig 4C, S2 Table). The numbers of down/upregulated pre-granulosa/Sertoli cell-specific genes increased gradually post-transplantation (Fig 4D), suggesting a potential involvement of these genes in this partial masculinization process.

Based on gene clustering analyses, the down- and upregulated genes were divided into three clusters (Fig 4E and 4F; Table 1, S3 Table). Within cluster I, several key pre-granulosa cell-specific genes such as *Fst*, *Bmp2*, *Wnt4*, and *Rspo1* [20,48,49] were downregulated in the ovarian grafts by day 10 post-transplantation (Fig 4G). GO terms in this cluster (Fig 4F) included “negative regulation of cell differentiation” (*Irx3*, *Axin2*, *Fst*, *Mbnl3*, *Nr0b1*, *Wnt4*, *Wnt9a*; p = 1.5E-03) and “reproductive structure development” (*Immp2l*, *Fgfr2*, *Fst*, *Nr0b1*, *Wnt4*; p = 1.2E-02). In clusters II and III, Sertoli cell-specific genes were upregulated starting from days 7–10 and 15–20 post-transplantation, respectively (Fig 4E and 4H; Table 1). GO terms associated with clusters II and III (Fig 4F) included “development of primary male sexual characteristics” (*Bik*, *Bcl2l11*, *Sox9*, *Dhh*, *Gjb2*, *Mtap7*, *Pdgfra*, *Tesc*; p = 3.1E-05) and “positive regulation of transcription” (*Glis3*, *Notch1*, *Pou3f3*, *Rora*, *Rgmb*, *Sox9*, *Ahr*, *Mef2c*, *Nr4a1*, *Nr4a2*, *Rel*, *Tesc*, *Tef*, etc.; p = 3.5E-02).

**Table 1:** Top 10 genes of the pre-granulosa cell-specific downregulated genes (cluster-I) and Sertoli cell-specific upregulated genes (cluster-II and III)^1)^.

Gene Symbol	Definition	4d	7d	10d ave.	15d	20d ave.	E12.5 ^2)^ XY > XX
**Cluster-I (downregulated on 10d)**							
*Cpa2*	carboxypeptidase A2, pancreatic	-1.6	-6.0	-15.4	-29.0	-48.8	-4.9
*Gm20265*		-1.6	-4.1	-13.2	-31.9	-30.5	-11.3
*Fam196b*	predicted gene, EG574403	-1.9	-3.2	-11.4	-21.4	-33.3	-30.6
*Mbnl3*	muscleblind-like 3 (Drosophila)	-1.9	-3.9	-6.9	-8.1	-15.2	-13.9
*Trim66*	tripartite motif-containing 66	-1.0	-2.0	-6.8	-9.0	-8.0	-5.7
*Gabrb1*	gamma-aminobutyric acid (GABA-A) receptor, subunit beta 1	-1.7	-3.3	-6.7	-9.3	-10.5	-16.7
*Col22a1*	collagen, type XXII, alpha 1	-1.4	-4.7	-6.1	-5.9	-8.3	-4.6
*Ppp1r3g*	protein phosphatase 1, regulatory (inhibitor) subunit 3G	-1.3	-3.5	-6.0	-6.2	-7.8	-3.8
*Igf2*	insulin-like growth factor 2	-1.6	-2.0	-5.7	-6.4	-13.6	-4.5
*Drp2*	dystrophin related protein 2	-1.5	-2.8	-5.1	-7.1	-8.5	-24.4
**Cluster-II (upregulated on 10d)**							
*Miox*	myo-inositol oxygenase	-1.0	-1.2	11.9	22.6	77.8	4.0
*Pla2g4c*	phospholipase A2, group IVC (cytosolic, calcium-independent)	-1.1	-1.3	8.5	23.8	24.9	3.1
*Rerg*	RAS-like, estrogen-regulated, growth-inhibitor	1.5	3.7	7.3	12.6	9.9	29.2
*Fam40b*	family with sequence similarity 40, member B	1.3	2.7	7.0	6.8	6.0	4.0
*Synm*	synemin, intermediate filament protein	1.8	2.5	6.6	10.4	7.8	6.7
*Zar1*	zygote arrest 1	-1.0	-1.2	6.5	12.3	13.0	5.1
*Gpm6b*	glycoprotein m6b	1.8	4.4	6.1	3.4	3.7	2.8
*Esrp1*	RNA binding motif protein 35A	-2.3	1.9	6.0	10.7	9.0	7.0
*Egflam*	EGF-like, fibronectin type III and laminin G domains	1.4	2.7	5.4	6.2	4.3	2.8
*Slc45a3*	solute carrier family 45, member 3	-1.4	-1.3	5.0	17.4	24.3	2.0
**Cluster-III (upregulated on 20d)**							
*Hao2*	hydroxyacid oxidase (glycolate oxidase) 3	1.4	1.3	1.2	1.5	10.4	4.2
*Aass*	aminoadipate-semialdehyde synthase	1.5	-1.1	1.4	2.4	10.2	7.6
*Crym*	crystallin, mu	-1.5	-1.3	-1.2	1.0	6.5	3.8
*Adhfe1*	alcohol dehydrogenase, iron containing, 1	1.2	-1.0	1.0	1.2	5.7	4.8
*Adh1*	alcohol dehydrogenase 1 (class I)	1.4	1.5	1.4	1.7	4.8	3.6
*Nceh1*	arylacetamide deacetylase-like 1	1.6	1.1	1.5	2.9	4.6	2.5
*Gjb2*	gap junction protein, beta 2	-1.1	-1.1	-1.0	1.1	4.3	28.9
*Cdo1*	cysteine dioxygenase 1, cytosolic	-1.3	-1.2	1.3	2.5	4.2	3.2
*Cage1*	cancer antigen 1	-1.1	1.0	1.9	3.7	3.8	3.1
*Mfsd2a*	major facilitator superfamily domain containing 2	-1.4	-1.5	1.4	3.2	3.5	4.1

^1)^ Fold changes of top 10 genes in cluster-I (pre-granulosa cell-specific genes downregulated on day 10 post-transplantation) and cluster-II and III (Sertoli cell-specific genes upregulated on days 10 and 20 post-transplantation) (also see Fig 4F).

2) Fold changes from a published dataset using *Sry*-EGFP Tg lines [47].

### Down- or upregulation of several key genes specific to pre-granulosa or Sertoli cells in ovarian grafts

**Fig 5:**
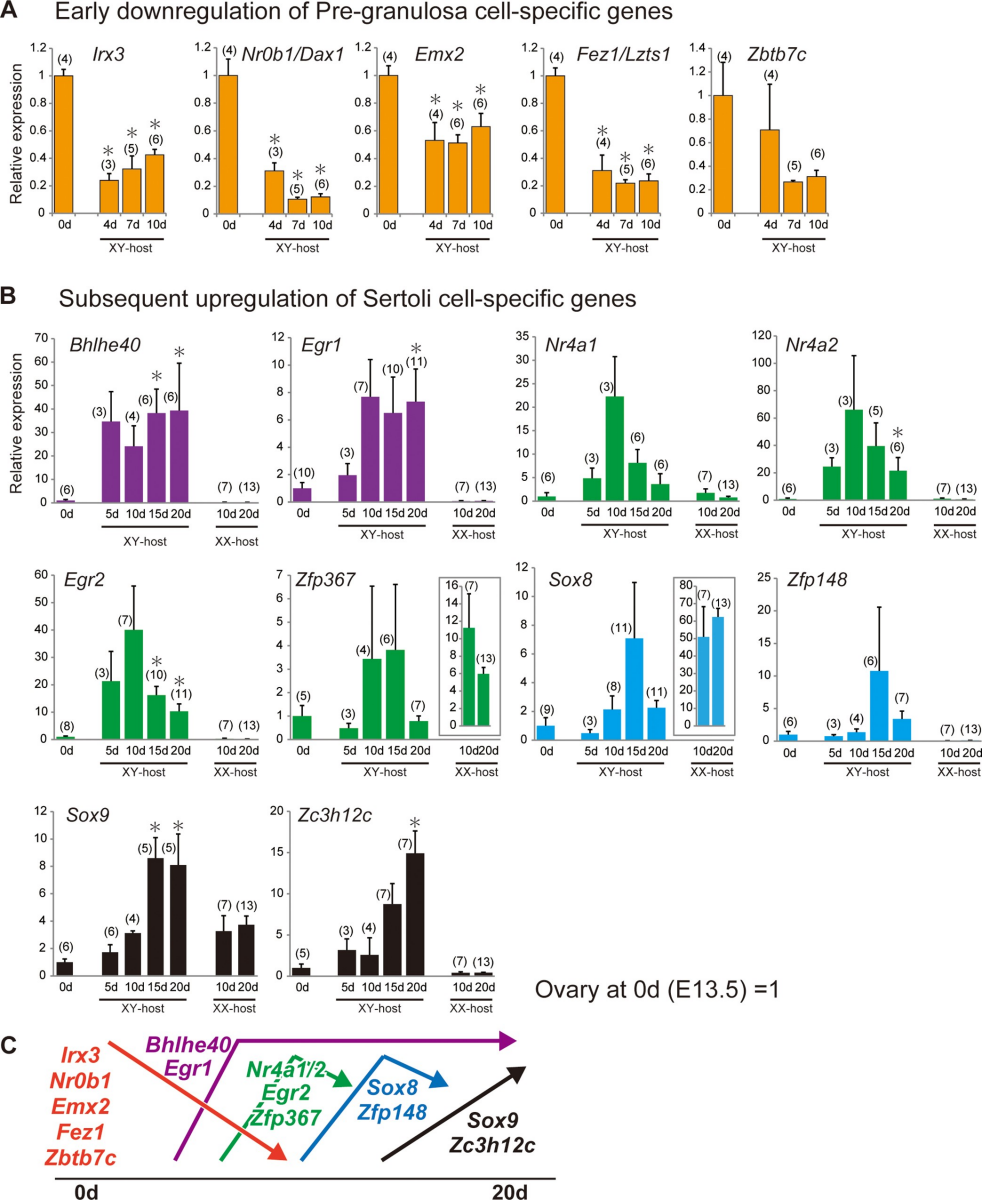
Temporal changes in the expression levels of each pre-granulosa/Sertoli cell-specific gene encoding a transcription/nuclear factor in grafted ovaries. **(A–B)** Bar graphs showing temporal changes in the expression levels (y-axis) of pre-granulosa (A) and Sertoli cell-specific genes (B) in ovarian grafts in male and female host mice on each day post-transplantation (x-axis). The data are expressed as means ± SEM. The expression levels on day 0 post-transplantation (i.e., the ovaries isolated from the 13.0-dpc embryos pretreated with busulfan) were set as 1.0 on the y-axis (*p<0.05 as compared with day 0, Dunnett’s test or Steel’s test). The numbers in parentheses indicate the number of explants used at each stage. **(C)** Expression profile categories for pre-granulosa or Sertoli cell-specific genes in grafted ovaries during partial masculinization.

By using a inducible SRY transgenic line, the first cellular sign of pre-granulosa cell differentiation in XX gonads was identified as the loss of SDSI during 11.5 to 11.75 dpc [4,17]. Moreover, in the present masculinization model, the re-acquisition of SDSI activity occurs in pre-granulosa cells, together with follicular degeneration, in the ovarian grafts on days 7–10 post-transplantation [17]. Among the genes that were downregulated by day 10 post-transplantation (including cluster I), we focused on transcription/nuclear factors that may be potentially involved in the re-acquisition of SDSI activity in pre-granulosa cells. Table 2 displays the 16 candidate downregulated genes encoding transcription/nuclear factors. RT-qPCR analyses of these genes in ovarian grafts of busulfan-treated mice confirmed significant downregulation of *Irx3* (*Iroquois related homeobox 3*, a granulosa cell-specific factor crucial for ovarian follicle development, together with a redundant function of *Irx5* [50]), *Nr0b1* (*Nuclear receptor subfamily 0*, *group B*, *member;* also known as *Dax1*), *Emx2* (*empty spiracles homeobox 2*; a crucial factor for the epithelial-to-mesenchymal transition of gonadal somatic cells [51]), and *Fez1*/*Lzts1* (*tumor suppressor gene 1*, which inhibits cell proliferation in ovarian cancer [52]), except for *Zbtb7c* (*zinc finger and BTB domain containing 7a*) (Fig 5A). All of these genes have been identified as ovarian genes that are independent of *Foxl2* action [18]. *Nr0b1*/*Dax1*, also known as anti-*Sry* gene [53,54], was also gradually downregulated in the ovarian grafts during the early phases of transplantation.

**Table 2:** List of the potentially 16 down- or 22 up-regulated genes of pre-granulosa or Sertoli cell-specific transcription/nuclear factor, respectively^1)^.

Gene Symbol	Definition	4d	7d	10d ave.	15d	20d ave.	E12.5 ^2)^ XY > XX
*Lhx9*	LIM homeobox protein 9	-7.6	-8.8	-10.4	-11.9	-14.2	-18.2
***Nr0b1***	nuclear receptor subfamily 0, group B, member 1	-1.9	-2.6	-4.6	-4.9	-6.5	-3.0
*Zim1*	zinc finger, imprinted 1	-1.4	-2.2	-4.3	-6.2	-8.7	-12.9
*Msx1*	homeobox, msh-like 1	-2.6	-3.0	-3.8	-6.1	-9.0	-10.2
***Irx3***	Iroquois related homeobox 3 (Drosophila)	-1.6	-1.8	-3.7	-5.3	-6.3	-21.2
*Dcaf12l1*	WD repeat domain 40B	-1.8	-2.9	-3.6	-4.1	-5.2	-7.8
***Emx2os***	EMX2OS mRNA, complete sequence	-1.5	-2.1	-3.3	-4.7	-5.4	-3.6
***Zbtb7c***	zinc finger and BTB domain containing 7C	-1.2	-1.6	-3.3	-3.6	-3.9	-4.0
*Prrx2*	paired related homeobox 2	-1.4	-1.9	-3.0	-2.8	-5.8	-5.7
*Lhx2*	LIM homeobox protein 2	-2.7	-3.0	-2.4	-1.9	-1.5	-2.0
*Zfp277*	zinc finger protein 277	-1.8	-2.2	-2.1	-2.1	-2.3	-5.0
*Limd2 ///* *LOC632329*	LIM domain containing 2	-1.0	-1.6	-2.0	-1.8	-1.9	-2.9
*Zfp612*	zinc finger protein 612	-1.1	-1.4	-1.9	-1.8	-2.0	-3.7
*Zfp503*	zinc finger protein 503	-1.8	-1.3	-1.8	-2.6	-2.3	-4.3
*Zfyve1*	zinc finger, FYVE domain containing 1	-1.2	-1.6	-1.7	-1.9	-2.4	-3.5
***Fez1***	fasciculation and elongation protein zeta 1 (zygin I)	1.1	-1.0	-1.3	-1.2	-1.7	-1.4
*Zbtb20*	zinc finger and BTB domain containing 20	3.4	7.8	9.9	12.6	17.5	3.6
***Egr2***	early growth response 2	10.0	8.2	6.9	203.4	10.4	3.3
***Bhlhe40***	basic helix-loop-helix family, member e40	2.8	2.8	2.9	13.0	5.1	2.2
*Klf6*	Kruppel-like factor 6	4.6	4.1	4.5	7.5	4.2	2.3
***Egr1***	early growth response 1	2.9	3.3	3.6	29.3	4.1	2.7
*Rora*	RAR-related orphan receptor alpha	1.8	1.6	2.1	2.8	3.9	2.6
***Sox8***	SRY-box containing gene 8	2.4	3.0	3.7	4.5	3.4	8.8
*Dtx4*	deltex 4 homolog (Drosophila)	1.2	1.6	2.0	1.9	2.9	2.1
*Glis3*	GLIS family zinc finger 3	1.5	1.2	1.6	1.4	2.8	7.2
***Zfp367***	zinc finger protein 367	1.2	1.5	2.7	2.7	2.3	3.5
*Znrf2*	zinc and ring finger 2	1.4	1.3	1.6	1.8	2.3	2.3
***Zc3h12c***	zinc finger CCCH type containing 12C	1.6	1.7	2.0	2.4	2.3	2.5
*Pdlim5*	PDZ and LIM domain 5	1.5	2.1	2.4	2.2	2.2	3.0
***Zfp148***	zinc finger protein 148	1.8	1.8	2.1	2.1	2.2	2.0
*Mef2c*	myocyte enhancer factor 2C	1.3	2.7	2.5	2.4	2.1	3.1
*Nr4a3*	nuclear receptor subfamily 4, group A, member 3	2.3	1.9	1.3	4.0	2.0	7.9
***Nr4a2***	nuclear receptor subfamily 4, group A, member 2	1.2	-1.1	1.3	1.9	1.6	5.5
*Zcchc11*	zinc finger, CCHC domain containing 11	1.1	1.7	1.9	1.9	1.5	2.4
*Zbtb1*	zinc finger and BTB domain containing 1	1.2	1.2	1.5	2.1	1.5	2.6
***Zc3h12c***	zinc finger CCCH type containing 12C	1.2	1.2	1.5	1.8	1.5	3.1
*Btbd3*	BTB (POZ) domain containing 3	1.1	1.2	1.5	1.5	1.5	3.1
***Nr4a1***	nuclear receptor subfamily 4, group A, member 1	1.2	-1.1	-1.1	6.5	1.2	2.2

^**1)**^ Fold changes of the selected transcription/nuclear factors down- or up-regulated in ovarian grafts at each stage (|fold| > 1.5).

^**2)**^ Fold changes from a published dataset [47].

Next, among the upregulated genes in ovarian transplants (including clusters II and III), we selected 22 transcription/nuclear factors showing differences in expression more than 1.5-fold at each stage (lower column in Table 2) in order to understand the molecular basis underlying the partial masculinization. RT-qPCR analyses of these genes revealed that *Bhlhe40* (*basic helix-loop-helix family*, *member e40*) and *Egr1* (*early growth response 1*) was upregulated on day 5–10 post-transplantation, and its high expression levels were maintained by day 20 post-transplantation. *Nr4a1* (*nuclear receptor subfamily 4*, *group A*, *member 1*), *Nr4a2*, *Egr2*, *Zfp367* (*zinc finger protein 367*), *Sox8* and *Zfp148*, albeit of no significance in some genes (e.g., *Nr4a1*, p = 0.08 on day 10; owing partly to the varying degrees of masculinization in each graft), displayed a transient upregulation pattern around on day 10–15 post-transplantation (Fig 5B). *Sox8* expression appeared to be upregulated 10–15 days post-transplantation, prior to significant *Sox9* upregulation at 15–20 days post-transplantation. This was consistent with our *Sox8 in situ* hybridization (Fig 3A and 3B). *Zc3h12c* gradually increased post-transplantation, with a significant peak on day 20. Moreover, such expression levels in the ovarian explants in the XY-host were compared with those in XX-host on days 10 and 20 post-transplantation (right two bars in Fig 5B). In the ovarian explants in the XX-host, most of the genes were maintained at lower levels in the ovarian grafts in XX-host than XY-host, except for *Zfp367* and *Sox8*, two gene transcripts that were previously shown to be enriched in the maturing oocyte [55,56]. Their high level expression in XX-host (see right graphs surrounded by a box in Fig 5B) may be possibly due to the presence of several growing antral follicles with the healthy oocytes survived even in the busulfan-treated samples in XX-host. The expression profile categories of these genes were schematically shown in Fig 5C.

### Upregulation of Androgen receptor (*Ar*) and its related genes in ovarian grafts in the male-host environment

**Fig 6:**
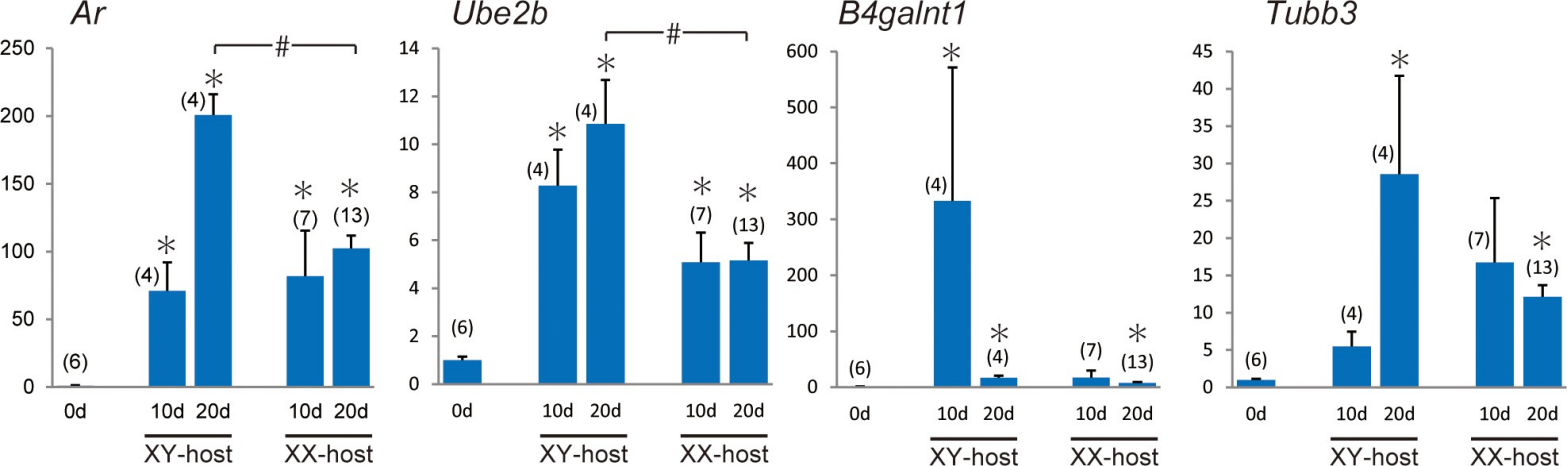
Expression profiles of AR and its target genes in grafted ovaries. Bar graphs showing temporal changes in the expression levels (y-axis) of *Ar* and AR-target genes, *Ube2b*, *B4galnt1* and *Tubb3*, in ovarian grafts in XY- and XX-host on each day post-transplantation (x-axis). The data are expressed as means ± SEM. The expression levels on day 0 post-transplantation were set as 1.0 on the y-axis (*p<0.05 as compared with day 0; #p<0.05 as compared between different host groups, Steel’s test). The numbers in parentheses indicate the number of explants used at each stage.

To examine the response to testosterone actions in ovarian grafts, we selected *Ar* (*Androgen receptor*) and AR-target genes in supporting cells such as *Ube2b* (*ubiquitin-conjugating enzyme E2B*, a direct target/mediator gene of AR in Sertoli cells [57]), *B4galnt1/Galgt1* (*beta-1*,*4-N-acetyl-galactosaminyl transferase 1*, a gene essential for proper spermatogenesis [58–60]), and *Tubb3* (*tubulin*, *beta 3 class III*, a direct target of AR in Sertoli cells [61]) among the upregulated genes (see S1 Table), and their expression levels were comparatively examined in the ovarian explants in the XY- and XX-hosts on days 10 and 20 post-transplantation (Fig 6). *Ar* and *Ube2b* were up-regulated in the ovarian explants of both intact male and female hosts on day 10 post-transplantation, and their expression in intact male hosts becomes higher than intact female hosts on day 20 post-transplantation. Moreover, expression levels of *B4galnt1* and *Tubb3* in the ovarian explants appear to be higher in XY-host than in XX-host on days 10 and 20 post-transplantation, respectively. These data suggest the positive responses of testosterone actions in the ovarian explants of XY-host in this masculinization model.

**Fig 7:**
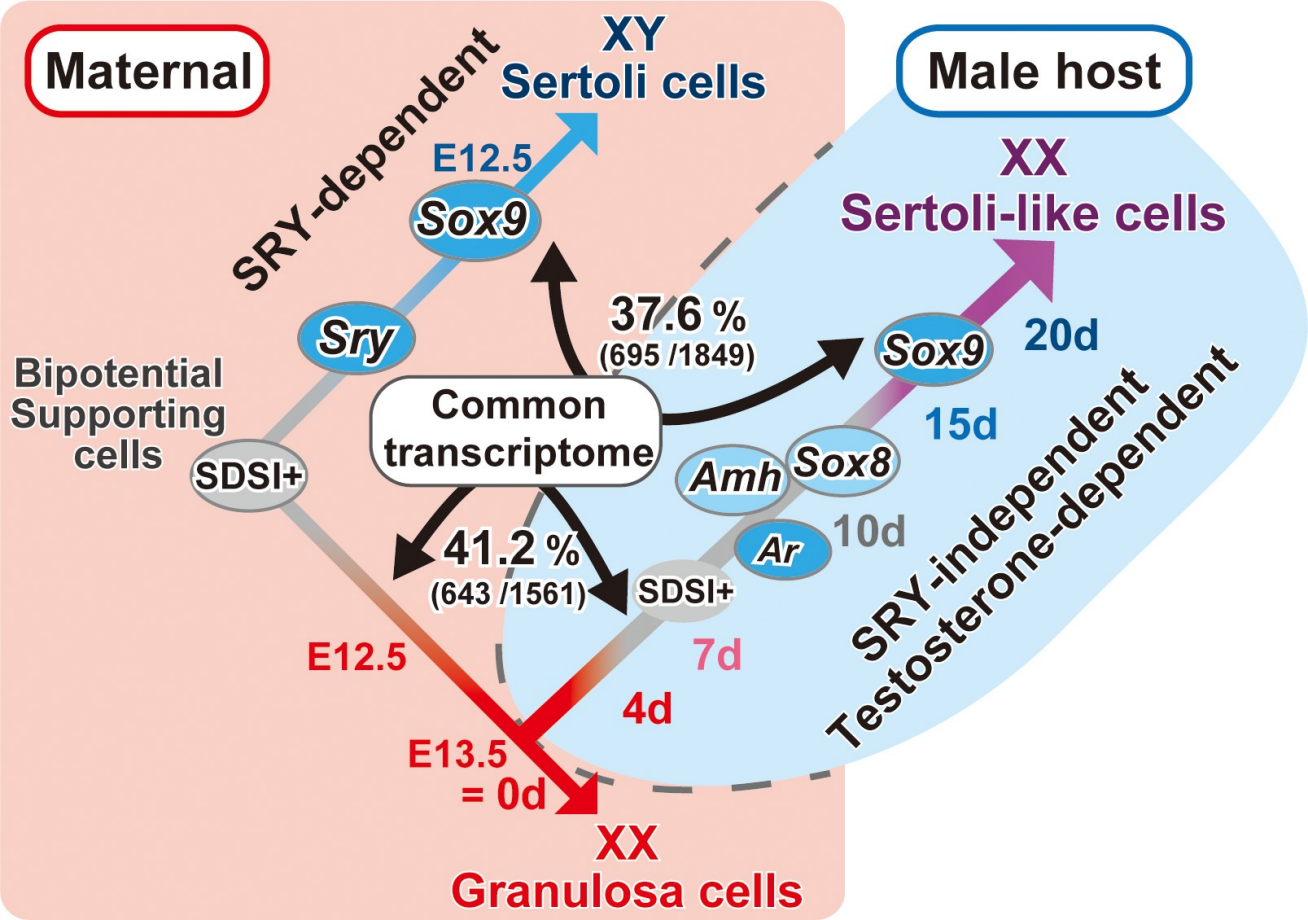
Testosterone-dependent masculinization of mouse fetal ovaries transplanted into the XY host. Schematic representation showing i) normal sex differentiation of SDSI-positive bipotential supporting cells in the maternal environment (pink background at left side) and ii) the partial masculinization of pre-granulosa cells, together with temporal upregulation of *Ar*, *Amh*, *Sox8* and *Sox9* in ovarian grafts in XY host (light blue background at right side). The global shared transcriptomes with Sertoli cell- and granulosa cell-specific genes between normal sex differentiation and masculinization processes was also shown (i.e., downregulation of 41.2% [643/1,561 gene probes] of the pre-granulosa cell-specific genes, versus upregulation of 37.6% [695/1,849 gene probes] of the Sertoli cell-specific genes [47]). Early downregulation of pro-ovarian genes and subsequent upregulation of pro-testis genes during days 4 to 20 post-transplantation (see Fig 5C) are indicated by a color change from red to purple in the bar. SDSI-positive bipotential state is also indicated as gray bars.

Finally, the global alteration of the shared transcriptomes with Sertoli cell- or granulosa cell-specific genes in the masculinization of ovarian grafts was schematically shown in Fig 7.

## Discussion

In fetal mouse ovarian explants grafted into male hosts, the masculinization processes are separated into distinct early (follicular degeneration/tubular structure formation) and late stages (subsequent appearance of ectopic SOX9-positive Sertoli-like cells in the ovarian grafts). Our temporal gene expression profiles of 4,747 downregulated and 6,730 upregulated genes in ovarian graft tissue post-transplantation revealed downregulation of 41.2% (643/1,561 gene probes) of the pre-granulosa cell-specific genes and upregulation of 37.6% (695/1,849 gene probes) of the Sertoli cell-specific genes (Figs 4C, 4D and 7). In particular, ovary-specific genes, including *Irx3*, *Nr0b1/Dax1*, *Emx2*, and *Fez1/Lzts1* [18,62,63], were downregulated within the first 10 days post-transplantation (Fig 5C). These four genes were previously identified as early ovarian genes that function independently of *Foxl2* action [18]. Our previous study demonstrated that under the same grafting conditions, SDSI was re-acquired in FOXL2-positive granulosa cells from degenerating follicles and tubular structures in ovarian grafts 7–10 days post-transplantation [17]. Furthermore, we found that loss of SDSI was the first sign of pre-granulosa cell differentiation at 11.5–11.75 dpc, during the onset of the female-specific expression of these four genes in differentiating ovaries. Collectively, these data suggest that downregulation of these candidate genes by day 7–10 post-transplantation may be positively associated with re-acquisition of SDSI in granulosa cells during partial masculinization of ovarian grafts.

In contrast to the early downregulation of ovarian genes, our RNA analyses suggested the transient upregulation profiles of *Nr4a1/2*, *Egr1/2*, *and Sox8* on days 10–15 post-transplantation, albeit not statistically significant in *Nr4a1* and *Sox8* (Fig 5C). It has previously been shown that the transcription factors *Nr4a1/2* and *Egr1/2*, albeit of their testis-specific expression at 12.5 dpc, are upregulated in the luteinization processes of granulosa cells in preovulatory ovarian follicles (Carletti and Christenson [64] and references therein). *Egr1*, a potential regulator of *Dmrt1* in Sertoli cells [65], was also shown to induce apoptosis in granulosa cells in atretic ovarian follicles [66]. Since follicle degeneration reaches a peak around 7–10 days post-transplantation [17], these data may suggest that these upregulated genes are involved in ovarian follicle degeneration and tubular formation in fetal ovarian grafts in a similar manner to the follicular atresia and luteinization in an estrous cycle of adult ovaries.

It has been demonstrated that *Nr4a1*, a positive regulator of GDNF expression in Sertoli cells [67], can upregulate AR expression in granulosa cells [68], further suggesting that it enhances AR-dependent signaling cascades during the degenerating follicles and subsequent partial masculinization. In previous reports, a granulosa cell-specific deletion of *Ar* gene showed that AR-mediated actions in the granulosa cells are required for the proper follicle dynamics [69,70], while excess androgen exposure in the fetal ovaries leads to prevalent hyperandrogenic infertility of polycystic ovary syndrome [71]. The present study demonstrated that the SOX9-positive Sertoli-like cells were ectopically induced not only in XY-host, but also in XX-host treated with testosterone/DHT. Especially, among three AR-target genes, *Ube2b* was significantly up-regulated in XY-cast (Fig 6), suggesting the positive correlation of *Ube2b* expression with this testosterone-dependent masculinization. However, it is also possible that, besides testosterone, other hormones (e.g., FSH and LH) derived from XY-host contribute to the follicular degeneration and subsequent masculinization along the hypothalamo-pituitary-gonadal axis [72–75]. Moreover, we could not completely rule out possible contribution of the side effects from transplantation (e.g., injury and wound healing) to this partial masculinization process. Therefore, the accurate function of each upregulated pro-testis gene (including *Ar* and AR-target genes) in this masculinization will require further genetic studies using fetal ovarian grafts with corresponding null mutations.

In the present study, *Sox8* is widely expressed in granulosa cells in degenerating follicles during both the AMH-positive and -negative stages in the tubular structures even at day 10 post-transplantation (Fig 3, S5 Fig). The present reverse genetic analysis showed that degenerating follicles were significantly more abundant in both *Sox8*^-/-^ and *Amh*^-/-^ ovarian explants on day 20 post-transplantation (Fig 3C, 3D and 3G), suggesting that a certain contribution of either *Sox8* or *Amh* activity resistance and/or delay of follicular degeneration in the ovarian grafts. This is consistent with the recent data showing the association between human primary ovarian insufficiency (POI) and *SOX8* variants [76], together with AMH as an ovarian reserve marker in POI (also see review by Visser et al. [77]). However, no appreciable change was detectable in the ectopic appearance of SOX9-positive Sertoli-like cells in either *Sox8*-null or *Amh*-null explants (Fig 3E, 3F and 3G). From these genetic data, we can conclude that these genes play a non-essential role in the later phase of this masculinization process, albeit that there still remains the possible redundant function with other factors in this process. This is because *Sox* genes such as *Sox8* and *Sox10* share target genes and have redundant functions in the regulation of *Sox9* expression during testis differentiation [9,14,16,42,78]. It is also possible that TGF-β signals in *Amh*-null ovarian grafts are functionally redundant, owing to the critical roles of other TGF-β family members (such as activin and nodal) in testis development.

Finally, in this masculinization model, SOX9-positive Sertoli-like cells are restricted to the presumptive medullary region 15–20 days post-transplantation (Fig 2A). One possible explanation is the existence of a specific subpopulation of pre-granulosa cells on the mesonephric side that continuously retains SDSI throughout the fetal stages in normal ovaries [17,21]. After birth, they are fated to contribute to the first wave of follicular growth in the postnatal ovarian development [21]. Since this SDSI-positive subpopulation may sustain a “ready-to-go” state of *Sox9* expression in normal developing ovaries, it is possible that such marginal subpopulation may contribute to this testosterone-dependent masculinization.

In conclusion, the present study reveals the transcriptome patterns of the testosterone-mediated masculinization in ovarian grafts in male nude mice. These gene expression profiles may help elucidate the infertility and masculinization of mammalian fetal ovaries caused by in utero exposure to inadequate hormonal conditions, as in the case of freemartin syndrome in mixed-sex cattle twins and polycystic ovary syndrome in mammals.

## Materials and methods

### Animals

All animal experiments in this study were carried out in strict accordance with the approved guidelines by the institutional committees for animal and recombinant DNA experiments at the University of Tokyo (UT) and Tokyo Medical and Dental University (TMDU). The procedures were approved by the Institutional Animal Care and Use Committee of UT (approval ID: P13-764, P15-049) and TMDU (0150259C2, 0160024C2, and 0170248C2).

Nude mice (8 weeks old; BALB/c, nu/nu, SLC, Japan) were used as host mice for the transplantation of fetal ovaries. 1 week prior to transplantation, some of the 7 weeks old nude mice were castrated and implanted subcutaneously with a 0.5–1.0 cm silicon tube with an internal diameter of 2 mm (Kaneka Medix Corp., Japan) containing testosterone (25 mg/ml; Wako, Japan), dihydrotestosterone (DHT; 100 mg/ml; Sigma-Aldrich), or sesame oil alone. In the present study, we used the almost same or higher dose of T/DHT than the previous experiments of androgen-treatment with silicon tube into mice [79–81]. The testosterone levels in each male host group were estimated as the weight of their seminal vesicles [36] (S1 Fig).

At 13.0 dpc, female embryos were obtained from pregnant wild-type females (C57BL/6 and ICR strain; SLC). For microarray and RT-qPCR analyses, all pregnant females were pretreated with busulfan (intraperitoneal injection, 40 mg/kg, twice at 10.5 and 11.5 dpc) to eliminate the influence of germ cells and their transcripts on the expression profiles in the grafts. We confirmed that the fetal ovaries of busulfan-treated mice could induce the appearance of testis cord-like structures with SOX9-positive Sertoli-like cells (S6 Fig) in a similar temporal manner to the non-treated ovarian grafts with oocytes [17].

The *Sox8*^-/-^ and *Amh*^-/-^ embryos were obtained by mating either heterozygous or homozygous females with the heterozygous male mice that were established by the CRISPR/Cas9 system (S3 and S4 Figs).

### Loss-of-function *Sox8* and *Amh* murine mutants generated using the CRISPR/Cas9 system

A pair of oligos targeting *Sox8* or *Amh* was annealed and inserted into the BsaI site of the pDR274 vector (Addgene), as described by Hashimoto and Takemoto [82]. The sequences of the oligos were as follows: 5’-GGGCCGCTTCACGTGTGGCT-3’ (*Sox8*) and 5’-TGCAGGGCTTACGTGCCGAG-3’ or 5’-CGTTGGGACACAGCGCTAGC-3’ (*Amh*). The vectors expressing *Cas9* mRNA and these guide RNAs targeting *Sox8* or *Amh* were introduced into fertilized eggs collected from C57BL/6 females. Several independent lines with frame-shift mutations in *Sox8* (17, 37 or 103 bp deletion just upstream of the first alpha helices of the HMG box domain, resulting in a complete loss of normal protein [83]) or *Amh* (26 or 35 bp deletion just upstream of the conserved sequences within the C-terminal domain, resulting in a complete loss of both cleavage REGR sequences and C-terminal TGF-beta-like domain [84]) were established by backcrossing C57BL/6 wild-type mice (S3A–S3C Fig). Since these independent lines had similar phenotypes, we used mainly the *Sox8* 17 bp and *Amh* 35 bp deletion lines for the transplantation experiments. *Sox8*^-/-^ and *Amh*^-/-^ mice exhibited the same phenotypes as described in previous reports [85–87]: both null females were fertile with functional ovaries, while *Sox8*^-/-^ males exhibited a typical phenotype of reduced Claudin-3 expression in the seminiferous tubules (S4A Fig), and *Amh*^-/-^ males showed retained uteruses (S4B Fig).

### Transplantation of fetal mouse ovaries

Transplantation of fetal ovaries was carried out using a previously reported procedure [17]. In brief, XX embryos at 13.0 dpc were collected from pregnant female mice. A pair of ovaries (without mesonephros) was isolated from the embryos in cold Dulbecco’s Modified Eagle’s Medium (DMEM; MERCK) with fine tweezers and needles. The 2 whole-ovaries of same pair were transplanted beneath one side of kidney capsule of host nude mice. Transplants were recovered from host kidneys 4–20 days post-transplantation. S4 Table shows the numbers of hosts and survival grafts in each experimental group.

### Histology and immunohistochemistry

The samples were fixed in 4% PFA-PBS at 4°C for 12 hours, dehydrated, and then embedded in paraffin. Serial sections at the horizontal level of each thin, flat graft (approximately 4 μm in thickness) were used for immunostaining of various markers as described below. The sections were incubated with anti-AMH (1:800 dilution; Santa Cruz), anti-DMRT1 (1:100 dilution; Santa Cruz), anti-FOXL2 (1:600 dilution; Abcam), anti-GATA4 (1:400 dilution; Santa Cruz), anti-GDNF (1:100 dilution; Santa Cruz), and anti-SOX9 (1:10,000 dilution; Merck Millipore) antibodies at 4°C for 12 hours. The reactions were visualized using Elite ABC Kit (Vector Laboratories), TSA Plus Biotin Kit (Perkin Elmer), or Alexa Fluor 488 Tyramide SuperBoost Kit (Thermo Fisher) with 4’, 6-diamidino-2-phenylindore (DAPI).

For morphometric analyses, anti-GATA4/AMH/SOX9-stained serial sectioning samples (every 10^th^ section from the horizontal sections) were photographed, and the numbers of normal healthy follicles, degenerating follicles derived from secondary or antral follicles, and SOX9-positive cells were counted in the sections with maximum areas. The degenerating follicle was estimated as the follicular structure except for a healthy follicle by the histological criteria such as an atretic oocyte and disarranged granulosa cells using the image data of the immunohistochemical and histological adjacent sections. The gonadal area (mm^2^) containing GATA4-positive cells was also measured using ImageJ 1.48V software (National Institutes of Health, Bethesda, MD, USA), and the relative numbers of follicles and SOX9-positive cells were estimated.

### *In situ* hybridization

All *in situ* hybridization experiments were performed using an RNAscope [88]. All samples were fixed in 10% (vol/vol) neutral buffered formalin at room temperature for 24 hours, dehydrated, and embedded in paraffin. Tissue sections were processed for RNA *in situ* detection using the RNAscope 2.0 High Definition-RED Kit according to the manufacturer’s instructions (ACDBio).

### Microarray processing and analysis

For microarray analyses, ovarian transplants initiated on 13.0 dpc (approximately 10 grafts at each stage per one microarray sample) were recovered on days 4, 7, 10, 15, and 20 post-transplantation from the host kidneys. Temporal fold changes in gene expression during each stage were examined and compared with those of 13.0 dpc ovaries pre-transplantation (i.e., on day 0 post-transplantation) (S1 Table).

Gene expression analysis was conducted using Mouse Genome 430 2.0 GeneChip arrays (Affymetrix). Total RNA from all samples was purified using the NucleoSpin RNA XS Kit (Macherey-Nagel); 100 ng purified total RNA were amplified and labelled with biotin, according to the GeneChip 3’ IVT Express Kit and GeneChip 3’ IVT PLUS Reagent Kit manuals (Affymetrix). The amplified biotinylated RNA (11 μg) was fragmented and hybridized to each array for 16 hours at 45°C in a rotating hybridization oven. Chips were washed and stained with streptavidin/phycoerythrin using the Fluidics Station 450 (Affymetrix) following protocol FS450_0001. Chips were scanned using the GeneChip 3000 Scanner (Affymetrix), and the outputs were obtained using the GeneChip Operating Software v. 1.4 (Affymetrix). All data are accessible via GEO Series accession number GSE88797.

Data were normalized using the robust multiarray algorithm [89], and further analyzed using the R software v 2.14.2 and the dChiP program (https://sites.google.com/site/dchipsoft/home). The averaged normalized values were used to derive fold changes in the duplicated raw data obtained from transplants on days 0, 10, and 20 post-transplantation. Among the altered genes (|fold| > 2), 4,747 were downregulated and 6,730 upregulated on day 4, 7, 10, 15, or 20 post-transplantation (S1 Table). The cell files (GSE18211) of Bouma et al. [47] were used to select 1,561 pre-granulosa and 1,849 Sertoli cell-specific genes at 12.5 dpc (|fold| > 2, p < 0.05; S2 Table, S3 Table) from the above altered gene pools. Gene functions were annotated based on gene ontology (GO) terms using DAVID Bioinformatics Resources 6.7 (http://david.abcc.ncifcrf.gov/).

### RT-PCR and quantitative RT-PCR

Total RNA was reverse-transcribed using random primers and the Superscript-III cDNA Synthesis Kit (Invitrogen). In order to confirm the shorter transcripts of mutated *Sox8* and *Amh* allele, we performed the RT-PCR analysis using the following primers: *Sox8* forward primer: 5’-TGT CAC ACG TGG AGG ATT CGG ACT C-3’, *Sox8* reverse primer: 5’-AGG GTC TTG CTG AGC TCT GCG TTA TGG AG-3’; *Amh* forward primer: 5’-ACG GAG AGG GAA CCT ATG CCG CTG-3’, *Amh* reverse primer: 5’-TGG CAG TTG TTG GCT TGG TAG GTC TC-3’ (their positions are indicated by red arrowheads in S3A and S3B Fig).

For Quantitative RT-PCR, Taqman gene expression probes for *Ar* (Mm00442688_m1), *Bhlhe40* (Mm00478593_m1), *B4galnt1/Galgt1* (Mm01135934_g1), *Egr1* (Mm00656724_m1), *Egr2* (Mm00456650_m1), *Emx2* (Mm00550241_ml), *Lzts1* (Mm01345507_m1), *Irx3* (Mm00500463_m1), *Nr0b1/Dax1* (Mm00431729_m1), *Nr4a1* (Mm01300401_m1), *Nr4a2* (Mm00443060_m1), *Sox8* (Mm00803423_m1), *Sox9* (Mm00448840_m1), *Tubb3* (Mm00727586_s1), *Ube2b* (Mm00494000_m1), *Zbtb7c* (Mm02375515_s1), *Zc3h12c* (Mm01177355_m1), *Zfp148* (Mm00711990_m1), and *Zfp367* (Mm00615562_m1) were purchased from Applied Biosystems. The relative levels of the transcripts were normalized against *Gapdh* (4352339E) levels as an endogenous reference.

### Statistical analysis

Normality of all quantitative data were analyzed by Shapiro-Wilk test prior to parametric or nonparametric test. As parametric test, the data were analyzed by one-way analysis of variance followed by Dunnett’s test. As non-parametric test, the data were analyzed by Kruskal–Wallis test followed by Steel’s test. Statistical significance was assessed at a threshold *p*-value of 0.05 or less.

## Supporting information

S1 Fig Relative seminal vesicle weight of normal host males and castrated host males treated with or without T, and DHT. A bar graph shows the relative seminal vesicle weight (seminal vesicle weight per gm body weight) of each host group. Compared to control (intact) male-host (XY-host, as set 100%), the relative seminal vesicle weight in other groups was approximately 11% in castrated host males (XY-cast-host), 59% in testosterone-treated castrated males (XY-cast-host +T), or 54% in 5α-Dihydrotestosterone-treated castrated males (XY-cast-host +DHT), respectively (means ± SEM, *p<0.05 as compared with non-treated host value in each host group; #p<0.05 as compared between two groups, Steel' test). The numbers in parentheses indicate the number of the host males examined after the transplants were recovered from their kidney capsule.
(TIF)

S2 Fig No SOX9-positive signals are detectable in the grafted ovaries even in the XY hosts on day 10 post-transplantation. Anti-SOX9 immunostaining of the wild-type ovarian tissues grafted into male (XY), female (XX), and castrated male (XY-cast) hosts, showing no ectopic SOX9-positive cells in all grafted ovaries on day 10 post-transplantation. Scale bars, 100 μm.
(TIF)

S3 Fig Establishment of independent lines of *Sox8*^-/-^ and *Amh*^-/-^ mice. **(A, B)** Amino acid sequences of wild-type and independent lines with frame-shift mutations of *Sox8* (17, 37, or 103 bp deletion just upstream of the first alpha helices of the HMG box domain, resulting in a complete loss of normal protein; A) and *Amh* (26 or 35 bp deletion just upstream of the conserved sequences within the C-terminal domain, resulting in a complete loss of both cleavage REGR sequences and C-terminal TGF-beta-like domain; B). The HMG box domain and C-terminal TGF-beta-like domain are shown in blue. Predicted amino acid sequences caused by frame-shift mutations are written in red (asterisk, stop codon). Red arrowheads show the positions of the RT-PCR primer sets (F, forward; R, Reverse), as shown in C. **(C)** RT-PCR analyses of the *Sox8* (left) or *Amh* (right) transcripts in the testes of wild-type (wild) and mutant (mut) males (2-month-old) by using the primer set that flanks the deleted mutation site (red arrowheads in a). The RT-PCR analyses confirm the presence of the only short (deleted) transcripts in each mutant testis. All blots are on the same gel. RT+ or RT- in each panel indicates the RT-PCR reaction samples treated with or without reverse transcriptase, respectively.
(TIF)

S4 Fig Phenotypic analysis of *Sox8*^-/-^ and *Amh*^-/-^ mice. **(A)** Anti-Claudin-3 (CLDN3; 1:1000 dilution; Thermo Fisher Scientific) immunostaining of the testes of wild-type and *Sox8*^-/-^ male mice (5-month-old), showing a typical *Sox8*-null phenotype of reduced CLDN3 expression (arrowheads) in the basal compartment of seminiferous tubules [82]. **(B)** Reproductive tracts of wild-type and *Amh*^-/-^ male mice at 3 months old, showing the presence of the uterus tubule (yellow arrow) that runs parallel to the vas deference (white arrow) in the *Amh*^-/-^ male. Scale bars, 20 μm.
(TIF)

S5 Fig AMH and *Sox8* signals in *Sox8*^-/-^ and *Amh*^-/-^ ovarian grafts. **(A)** Anti-AMH immunostaining of wild-type (on day 10 post-transplantation) and *Sox8*^-/-^ (on day 20 post-transplantation) ovarian grafts, showing AMH-positive signals are properly seen in the follicles of *Sox8*^-/-^ grafted ovarian tissues. **(B)** *In situ* hybridization using a *Sox8* antisense probe of wild-type and *Amh*^-/-^ ovarian grafts on day 20 post-transplantation, showing no appreciable differences of *Sox8*-positive signals in the degenerating follicles (arrowheads) between wild-type and *Amh*^-/-^ ovarian grafts. Scale bars, 100 μm.
(TIF)

S6 Fig Ectopic appearance of SOX9-posititve Sertoli cell-like cells in grafted ovaries of busulfan-treated mice transplanted into male host mice. Anti-AMH or SOX9 immunostaining of ovarian tissues of busulfan-treated wild-type mice grafted into male host mice on days 10 (upper) and 20 (lower) post-transplantation. Typical testis cord-like structures (arrows in insets) and ectopic appearance of SOX9-positive cells in the medullary region were detected in busulfan-treated grafted ovaries, similarly to non-treated grafted ovaries. The low-magnification images of each ovarian tissues are shown in the insets at the upper right corner. Scale bars, 100 μm.
(TIF)

S1 Table The list of the whole down/upregulated genes and their GO terms in the ovarian grafts. S2 Table A/B show the fold changes of 4,747 down/6,730 upregulated genes in the ovarian grafts at each stage (|fold| > 2). S1 Table C/D show the lists of GO terms (p < 0.01) of the down/upregulated genes (|fold| > 2, false discovery rate [q] < 0.3), respectively. S1 Table E shows lists of gene content in the selected GO terms from S1C and S1D Table.
(XLSX)

S2 Table The list of the down/upregulated pre-granulosa/Sertoli cell-specific genes in the ovarian grafts. S2A and S2B Table show the expression fold changes of 695 down/643 upregulated pre-granulosa/Sertoli cell-specific genes in the ovarian grafts at each stage. S2C and S2D Table show the expression fold changes of 1,561 pre-granulosa/1,849 Sertoli cell-specific gene expression in the ovarian grafts.
(XLSX)

S3 Table The list of Cluster-I, II, and III and their GO terms. S3A Table shows cluster-I genes, which are downregulated pre-granulosa cell-specific genes, and S3B and S3C Table show clusters-II/III genes, which are upregulated Sertoli cell-specific genes in ovarian grafts. Cluster-I is the 189 pre-granulosa cell-specific genes downregulated in grafted ovaries on day 10 post-transplantation (|fold| > 2). Cluster-II is the 141 Sertoli cell-specific genes upregulated in grafted ovaries, especially on day 10 post-transplantation (|fold| > 2). Cluster-III is the other 261 Sertoli cell-specific genes upregulated in grafted ovaries on day 20 post-transplantation (|fold| > 2). S3D and S3E Table show the lists of GO terms of the genes in clusters-I (S3A Table) and clusters-II/III (S3B and S3C Table). S3F Table shows lists of gene content in the selected GO terms from S3D and S3E Table.
(XLSX)

S4 Table The numbers of host mouse, donor litter, donor ovary, and obtained ovarian graft in the transplantation experiment. (XLSX)
