# Supplementary figures and images for: Molecular and genetic characterization of partial masculinization in embryonic ovaries grafted into male nude mice
Source: PLoS One. 2019 Mar 6;14(3):e0212367. doi: 10.1371/journal.pone.0212367 (PMC6402656; doi:10.1371/journal.pone.0212367)

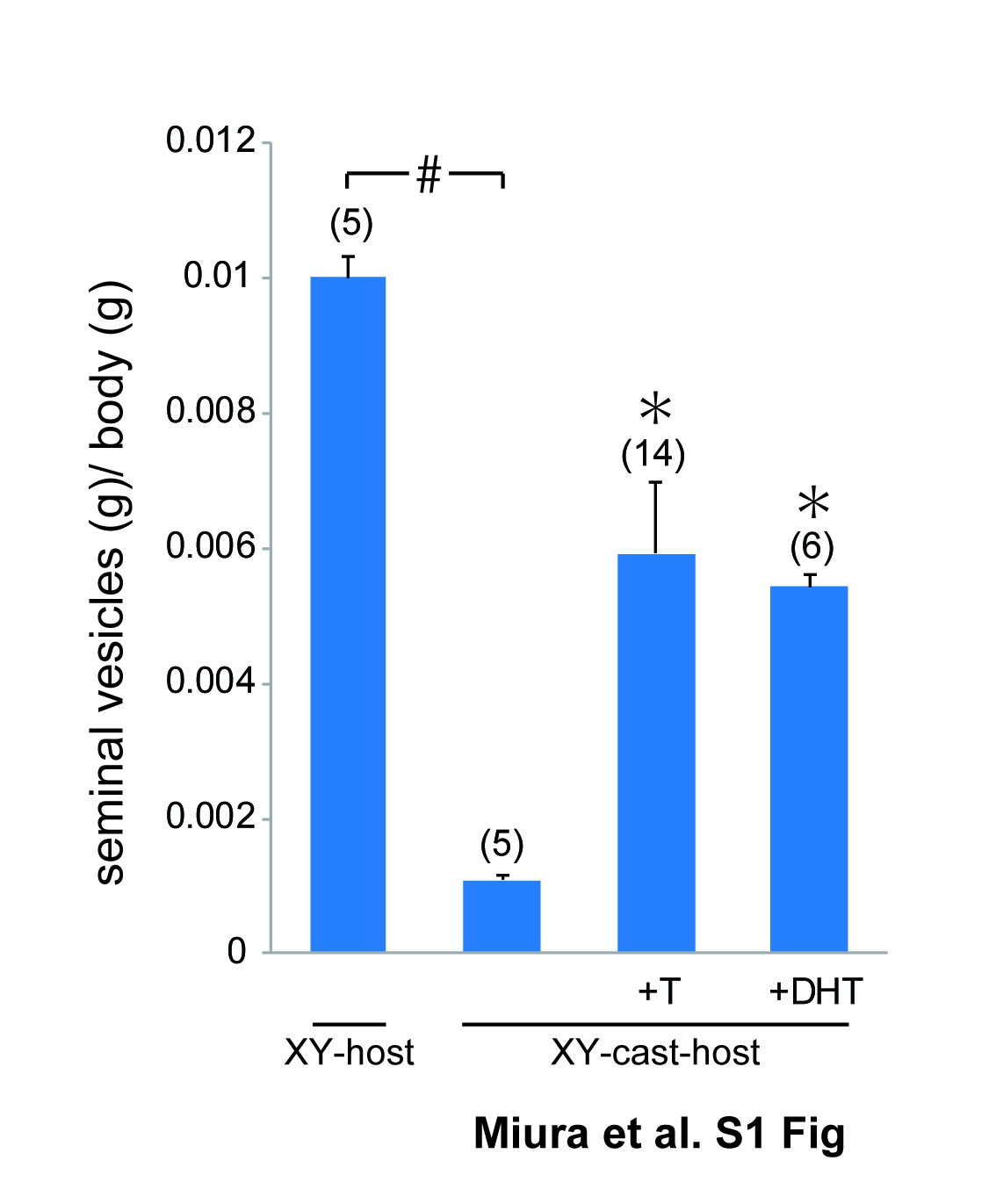

Supplement: S1 Fig — A bar graph shows the relative seminal vesicle weight (seminal vesicle weight per gm body weight) of each host group. Compared to control (intact) male-host (XY-host, as set 100%), the relative seminal vesicle weight in other groups was approximately 11% in castrated host males (XY-cast-host), 59% in testosterone-treated castrated males (XY-cast-host +T), or 54% in 5α-Dihydrotestosterone-treated castrated males (XY-cast-host +DHT), respectively (means ± SEM, *p<0.05 as compared with non-treated host value in each host group; #p<0.05 as compared between two groups, Steel' test). The numbers in parentheses indicate the number of the host males examined after the transplants were recovered from their kidney capsule. (TIF) [file pone.0212367.s001.tif]

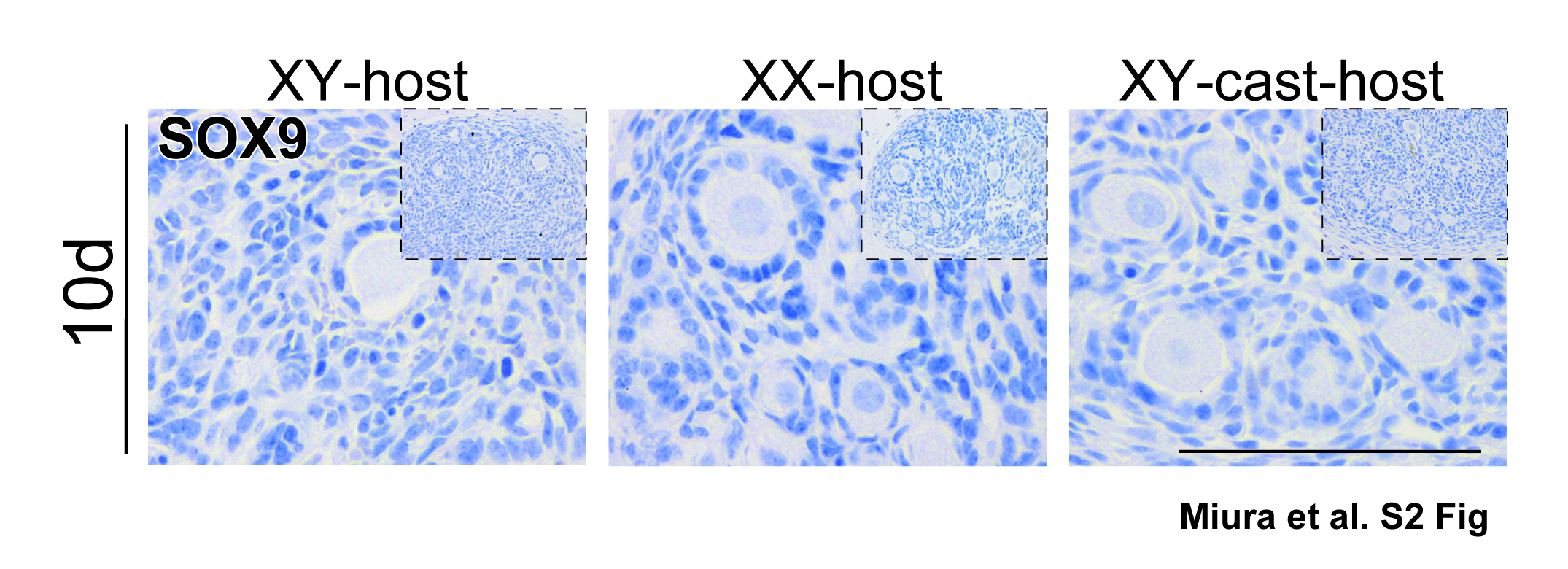

Supplement: S2 Fig — Anti-SOX9 immunostaining of the wild-type ovarian tissues grafted into male (XY), female (XX), and castrated male (XY-cast) hosts, showing no ectopic SOX9-positive cells in all grafted ovaries on day 10 post-transplantation. Scale bars, 100 μm. (TIF) [file pone.0212367.s002.tif]

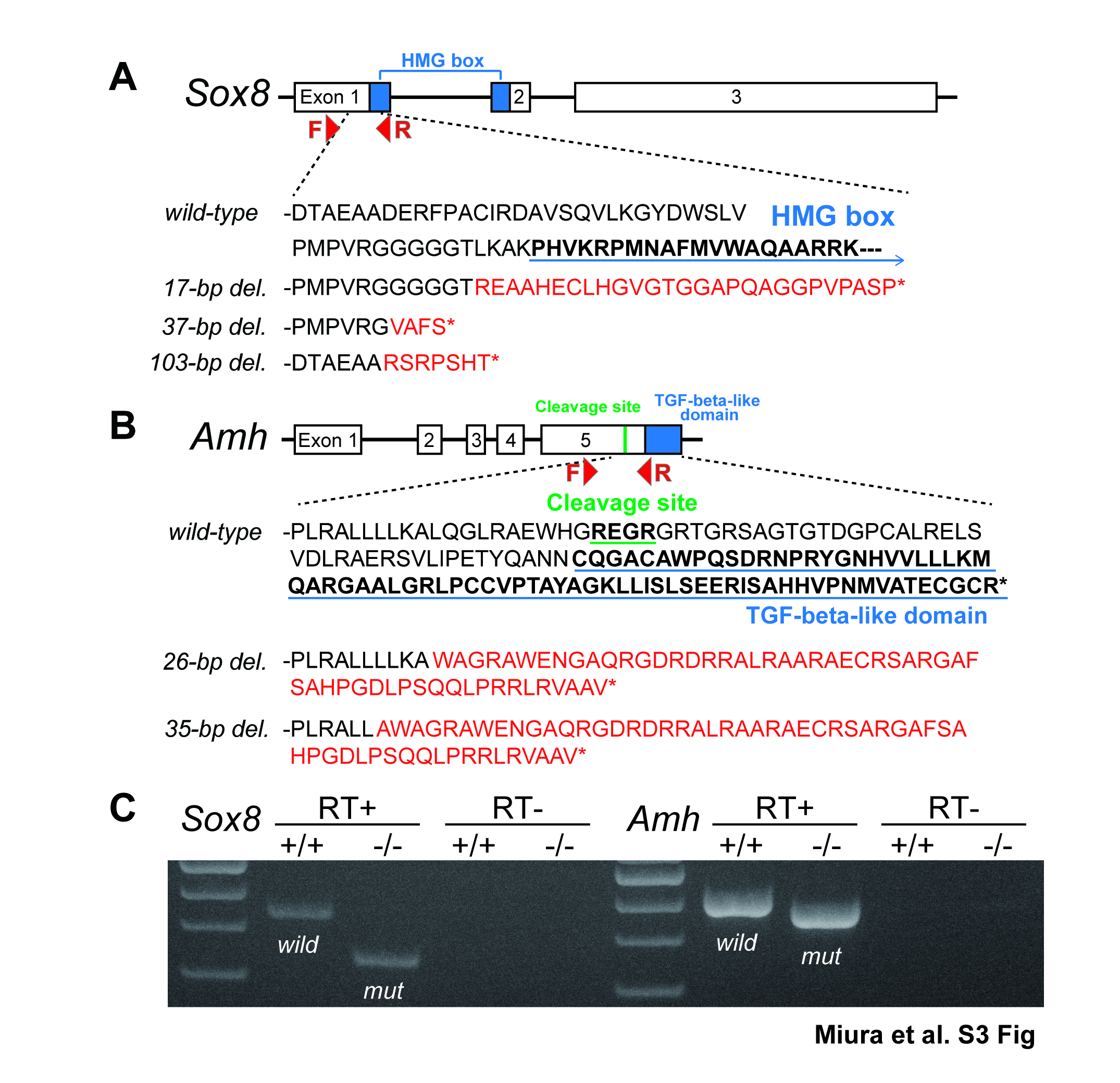

Supplement: S3 Fig — (A, B) Amino acid sequences of wild-type and independent lines with frame-shift mutations of Sox8 (17, 37, or 103 bp deletion just upstream of the first alpha helices of the HMG box domain, resulting in a complete loss of normal protein; A) and Amh (26 or 35 bp deletion just upstream of the conserved sequences within the C-terminal domain, resulting in a complete loss of both cleavage REGR sequences and C-terminal TGF-beta-like domain; B). The HMG box domain and C-terminal TGF-beta-like domain are shown in blue. Predicted amino acid sequences caused by frame-shift mutations are written in red (asterisk, stop codon). Red arrowheads show the positions of the RT-PCR primer sets (F, forward; R, Reverse), as shown in C. (C) RT-PCR analyses of the Sox8 (left) or Amh (right) transcripts in the testes of wild-type (wild) and mutant (mut) males (2-month-old) by using the primer set that flanks the deleted mutation site (red arrowheads in a). The RT-PCR analyses confirm the presence of the only short (deleted) transcripts in each mutant testis. All blots are on the same gel. RT+ or RT- in each panel indicates the RT-PCR reaction samples treated with or without reverse transcriptase, respectively. (TIF) [file pone.0212367.s003.tif]

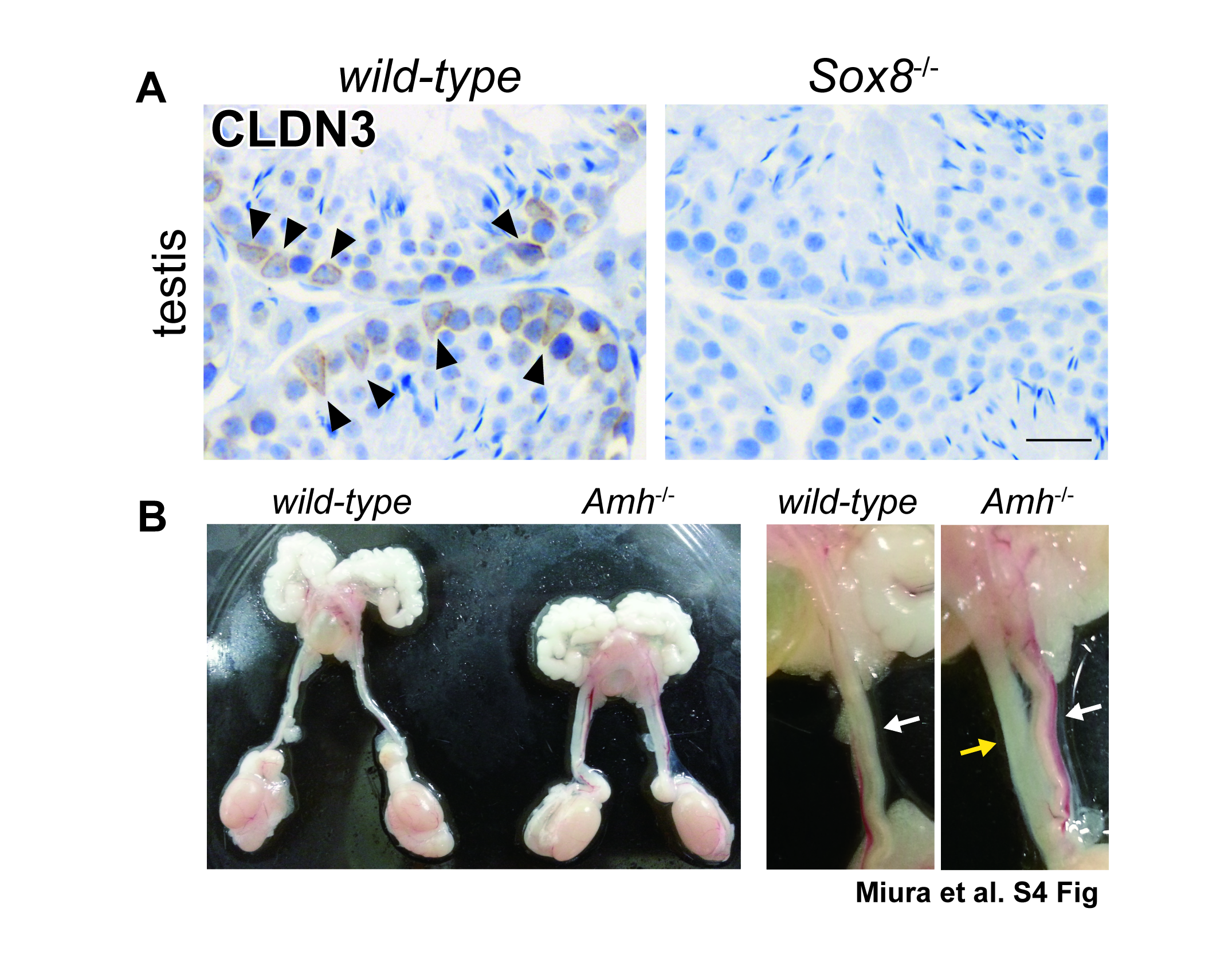

Supplement: S4 Fig — (A) Anti-Claudin-3 (CLDN3; 1:1000 dilution; Thermo Fisher Scientific) immunostaining of the testes of wild-type and Sox8-/- male mice (5-month-old), showing a typical Sox8-null phenotype of reduced CLDN3 expression (arrowheads) in the basal compartment of seminiferous tubules [82]. (B) Reproductive tracts of wild-type and Amh-/- male mice at 3 months old, showing the presence of the uterus tubule (yellow arrow) that runs parallel to the vas deference (white arrow) in the Amh-/- male. Scale bars, 20 μm. (TIF) [file pone.0212367.s004.tif]

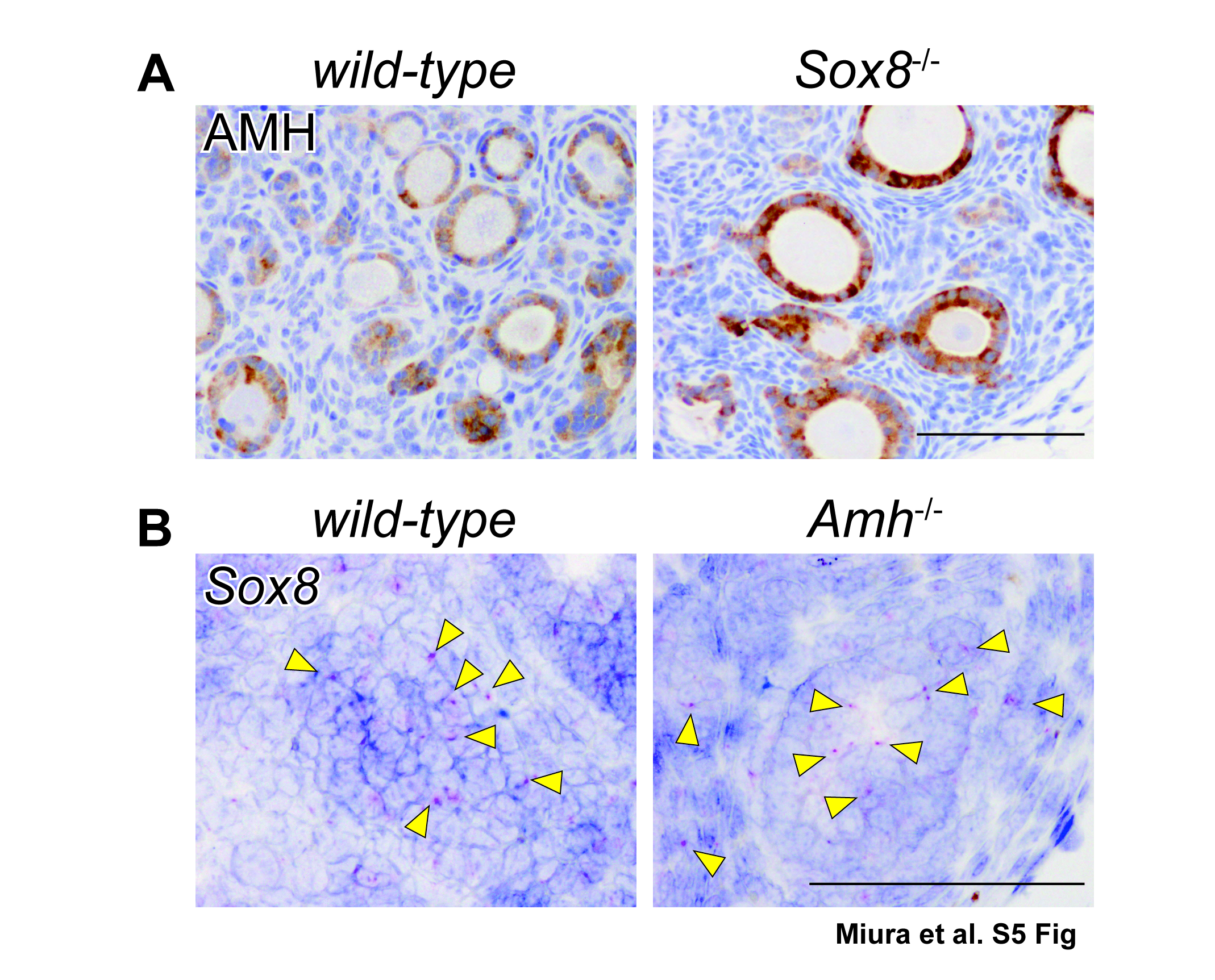

Supplement: S5 Fig — (A) Anti-AMH immunostaining of wild-type (on day 10 post-transplantation) and Sox8-/- (on day 20 post-transplantation) ovarian grafts, showing AMH-positive signals are properly seen in the follicles of Sox8-/- grafted ovarian tissues. (B) In situ hybridization using a Sox8 antisense probe of wild-type and Amh-/- ovarian grafts on day 20 post-transplantation, showing no appreciable differences of Sox8-positive signals in the degenerating follicles (arrowheads) between wild-type and Amh-/- ovarian grafts. Scale bars, 100 μm. (TIF) [file pone.0212367.s005.tif]

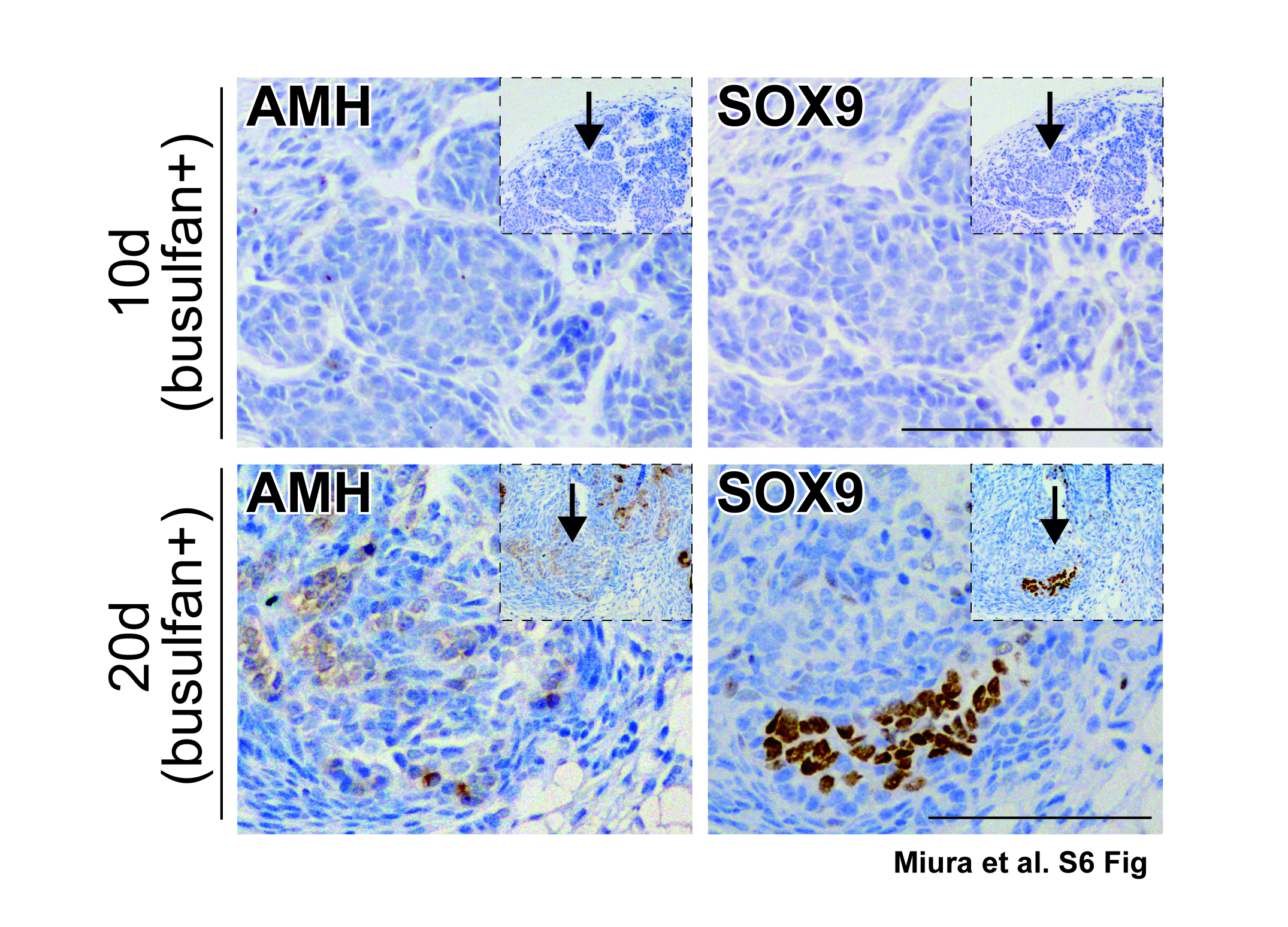

Supplement: S6 Fig — Anti-AMH or SOX9 immunostaining of ovarian tissues of busulfan-treated wild-type mice grafted into male host mice on days 10 (upper) and 20 (lower) post-transplantation. Typical testis cord-like structures (arrows in insets) and ectopic appearance of SOX9-positive cells in the medullary region were detected in busulfan-treated grafted ovaries, similarly to non-treated grafted ovaries. The low-magnification images of each ovarian tissues are shown in the insets at the upper right corner. Scale bars, 100 μm. (TIF) [file pone.0212367.s006.tif]
